# Assembly factor for spindle microtubules (*ASPM*) promotes osimertinib resistance in lung cancer by increasing *EGFR* stability

**DOI:** 10.3389/fgene.2025.1593314

**Published:** 2025-09-05

**Authors:** Pengpeng Gui, Zhengyi Han, Zhenyu Yin, Peng Cao, Xin Zhou, Yan Li

**Affiliations:** ^1^ State Key Laboratory of Technologies for Chinese Medicine Pharmaceutica Process Control and intelligent Manufacture, Nanjing University of Chinese Medicine, Nanjing, China; ^2^ Nanjing Drum Tower Hospital Clinical College of Nanjing University of Chinese Medicine, Nanjing, China; ^3^ Institute of Elderly Care Services and Management, Nanjing University of Chinese Medicine, Nanjing, China; ^4^ Department of Oncology, First Affiliated Hospital of Nanjing Medical University, Nanjing, China; ^5^ Department of Oncology, The Affiliated Suqian First People’s Hospital of Nanjing Medical University, Suqian, China

**Keywords:** non-small cell lung cancer (NSCLC), assembly factor for spindle microtubules (ASPM), tumor invasiveness, osimertinib, EGFR-TKI resistance, EGFR mutation

## Abstract

**Background:**

Non-small cell lung cancer (NSCLC) represents approximately 85% of all lung malignancies, with lung adenocarcinoma (LUAD) being the predominant histologic subtype. Epidermal growth factor receptor (*EGFR*) mutations serve as critical therapeutic targets in NSCLC; however, resistance to *EGFR* tyrosine kinase inhibitors (*EGFR*-TKIs) remains a major clinical challenge. Recent studies highlight the need to identify molecular drivers of resistance to improve therapeutic outcomes.

**Method:**

This study analyzed tumor tissue datasets to investigate the role of the assembly factor for spindle microtubules (*ASPM*) in NSCLC progression and drug resistance. Bioinformatics methods revealed high expression of *ASPM* in tumor tissues and its association with low patient survival. Functional validation was performed using the *EGFR*-TKI-resistant cell line PC9 osimertinib-resistant (PC-9 OR), with *ASPM*-silenced models. Cellular proliferation, invasion, and EGFR protein stability analyses were conducted. Additionally, the therapeutic impact of *ASPM* silencing and overexpression combined with the third-generation TKI osimertinib was evaluated.

**Results:**

*ASPM* is significantly upregulated in NSCLC tumor tissues and is strongly associated with reduced patient survival. *ASPM* silencing attenuates PC-9 and PC-9 OR malignant phenotypes, including proliferation and invasion, and sensitizes resistant cells to osimertinib. In addition, inhibiting the expression of *ASPM* effectively reduces damage to the cell cycle and protein stability of drug-resistant cells, thereby restoring the expression and function of *EGFR*.

**Conclusion:**

This study identified *ASPM* as a novel regulator of *EGFR*-TKI resistance in NSCLC, with dual roles in promoting tumor aggressiveness and stabilizing *EGFR* signaling. Targeting *ASPM* may represent a promising therapeutic strategy to overcome *EGFR*-TKI resistance, enhance osimertinib efficacy, and expand treatment options for refractory NSCLC patients. These findings provide a foundation for developing *ASPM*-directed therapies in precision oncology.

## Highlights

1. *ASPM* is highly expressed in lung cancer tissues.
2. *ASPM* significantly enhanced the proliferation and invasion of drug-resistant LUAD cells.
3. *ASPM* plays a key role in regulating the cell cycle and drug resistance of drug-resistant LUAD cells.

## 1 Introduction

Lung cancer remains the main killer worldwide (Guo et al., 2021; Guo et al., 2022; Li and Qiao, 2022). Non-small cell lung cancer (NSCLC), a common lung malignancy that accounts for approximately 85% of all lung cancers (Hirsch et al., 2017; Yao et al., 2022; Zeng et al., 2023), is the leading cause of cancer-related death worldwide (Thai et al., 2021). Lung adenocarcinoma (LUAD) is the most common subtype of NSCLC (Hirsch et al., 2017). Epidermal growth factor receptor (*EGFR*) (Yu et al., 2024; Dai et al., 2025; Xia et al., 2025) mutations are pivotal therapeutic targets (Hendriks et al., 2024). However, most of these patients develop resistance after a period of *EGFR* -tyrosine kinase inhibitor (TKI) therapy (Wu and Shih, 2018; Dai et al., 2025; Xia et al., 2025), in which *EGFR* mutations and *EGFR* overexpression are among the main causes of *EGFR*-TKI resistance (Karlsen et al., 2021; Zhou et al., 2021; Tian et al., 2022; Blaquier et al., 2023). Therefore, determining genes that can affect cellular drug resistance as well as the stability of the EGFR protein is highly important for the treatment of NSCLC (Herbst et al., 2018; Tian et al., 2022).

Originally discovered as a centrosomal protein that regulates neurogenesis and brain size, assembly factor for spindle microtubules (ASPM) has been shown to be relevant in a variety of cellular processes, such as mitosis, the cell cycle, protein stability, and the repair of DNA double-strand breaks (DSBs), in the last few years (Capecchi and Pozner, 2015; Tsai et al., 2023). Currently, *ASPM* has been shown to play a role in a variety of cancers, including pancreatic, gastric, and cholangiocarcinomas (Hsu et al., 2019; Hsu et al., 2021; Chang et al., 2024). However, it remains unclear whether the *ASPM* gene influences drug resistance in LUAD.

In this study, *ASPM* was found to be highly expressed in NSCLC by bioinformatics analysis and correlated with poor patient prognosis. The results revealed that a high level of *ASPM* expression was significantly and positively correlated with the proliferation and invasion ability of tumor cells. By silencing the *ASPM* gene, the proliferation and invasive activity of tumor cells can be effectively inhibited. Second, silencing the *ASPM* gene could significantly increase the sensitivity of drug-resistant cells to the third-generation *EGFR*-TKI drug osimertinib, and more importantly, *ASPM* was highly expressed in *EGFR*-TKI-resistant cell lines and played a key role in cellular drug resistance by affecting the stability of the EGFR protein. These findings suggest that *ASPM* may be a potential target for reversing drug resistance.

## 2 Materials and methods

### 2.1 Patient samples

Patient samples were obtained from the specimen bank of the central laboratory of Nanjing Medical University (Nanjing, China). The study was conducted in accordance with the ethical principles of the Declaration of Helsinki (2013 revision) and was approved by the Hospital Institutional Review Board (approval number: 2017-SRFA-104). Written informed consent was obtained from all participants before sample collection.

### 2.2 Cell culture

Lewis lung carcinoma (LLC), PC-9 and PC-9 OR cells were purchased from the Cell Bank of the Chinese Academy of Sciences (https://www.cellbank.org.cn/eindex.php). LLC was cultured in Dulbecco’s modified Eagle’s medium (DMEM) supplemented with 10% fetal bovine serum (FBS) (VivaCell, Shanghai, China) and 1% penicillin‒streptomycin. PC-9 cells and PC-9 OR cells were cultured in RPMI 1640 supplemented with 10% fetal bovine serum (VivaCell, Shanghai, China) and 1% penicillin‒streptomycin.

### 2.3 Small interfering RNA (siRNA) assay

According to the sequences in Table 1, siRNAs purchased from GenScript were designed to target both the *ASPM*-1 and *ASPM*-2 sites in *ASPM*. Target cells were cultured to the appropriate density (usually 70%–80% confluence), siRNA was transfected into cells via the liposome-mediated transfection method lipo3000 (Thermo Fisher Scientific, Massachusetts, America), and after 6 h of incubation in Opti-MEM (Thermo Fisher Scientific, Massachusetts, America), the cells were switched to normal medium, and the cells were cultured for 24 h. RNA was extracted, and the target gene mRNA was detected via real-time quantitative PCR (qPCR) to detect the mRNA levels of target genes to assess the silencing efficiency of the siRNAs.

**TABLE 1:** Sequence of the siRNAs.

Gene	Forward/Reverse	Primer sequence (5′ to 3′)
*ASPM*-1	Sense (5′-3′)	5′-GCU​UGC​AAU​ACA​GCA​AUA​ATT-3′
	Antisense (5′-3′)	5′-UUA​UUG​CUG​UAU​UGC​AAG​CTT-3′
*ASPM*-2	Sense (5′-3′)	5′-GCA​GCA​UGC​CGU​UUG​UUU​ATT-3′
	Antisense (5′-3′)	5′-UAA​ACA​AAC​GGC​AUG​CUG​CTT-3′

### 2.4 CCK8 cell proliferation assay

LLC, PC-9, and PC-9OR cells were inoculated in 96-well plates at a density of 3,000 cells per well and incubated at 37 °C and 5% CO2. 0, 12, 24, and 48 h later, 10 μL of CCK8 reagent was added to each well and incubated for 1 h. Cell proliferation was measured by measuring the absorbance at 450 nm via an enzyme marker (Wu et al., 2022).

### 2.5 Transwell cell migration assay

The cells were digested with trypsin and prepared into single-cell suspensions via Opti-MEM, and the cell concentration was adjusted to 1.5 × 10^5^ cells/ml. Transwell chambers with polycarbonate membranes with an 8 μm pore size were selected, and the chambers were placed into 24-well plates to ensure that the chambers fit tightly into the well plate. We added 100 µL of cell suspension to the upper chamber of the Transwell system and added medium containing 10% fetal bovine serum to the lower chamber to promote cell migration; thereafter, the Transwell system was incubated in an incubator for 72 h. We gently rinsed the Transwell system with PBS, fixed the cells with fixative (4% paraformaldehyde) for 30 min, and stained the cells with crystal violet stain for 30 min. Thereafter, we stained the cells for 30 min and used a microscope to observe the cells that moved to the bottom of the membrane. Typically, 10 random fields of view were selected for imaging.

### 2.6 RNA extraction and RT‒qPCR

RNA was extracted via the TRIzol (Kyoto, Japan, Takara Bio) method (Rio et al., 2010), and the RNA concentration was detected via a quantitative nucleic acid analyzer. The RNA was reverse transcribed into cDNA with a reverse transcription kit (Vazyme, Hiscript' IIRTMix R323.), and changes in the RNA expression levels of the *ASPM* and *EGFR* genes were detected via RT‒qPCR via the chimeric fluorescence method in cells and tissues (Vazyme, Taq Pro Universal SYBR qPCR Master Mix Q712.). All primer sequences are shown in Table 2. All PCRs were repeated 6 times, and the results were calculated via the 2^−ΔΔCt^ method and calibrated with GraphPad Prism 9.5 (GraphPad Software).

**TABLE 2:** Sequences of the RT‒PCR primers.

Gene	Forward/Reverse	Primer sequence (5′ to 3′)
*ASPM*	Forward	GGC​CCT​AGA​CAA​CCC​TAA​CGA
	Reverse	AGC​TTG​GTG​TTT​CAG​AAC​ATC​A
*EGFR*	Forward	AGG​CAC​GAG​TAA​CAA​GCT​CAC
	Reverse	ATG​AGG​ACA​TAA​CCA​GCC​ACC
GAPDH	Forward	GGA​GCG​AGA​TCC​CTC​CAA​AAT
	Reverse	GGC​TGT​TGT​CAT​ACT​TCT​CAT​GG

### 2.7 Western blot

Proteins were extracted from cell and tissue samples via a RIPA kit (Shanghai, China; Beyotime). Proteins were separated by SDS‒PAGE (7.5% sodium dodecyl sulfate‒polyacrylamide gel electrophoresis) and transferred to polyvinylidene difluoride (PVDF) membranes. The membrane was blocked with 5% skim milk for 60 min and incubated with primary antibodies, including ASPM (Proteintech, 26223-1-AP) (1:600) and EGFR (Cusabio, CSB-PA005571LA01HU) (1:1000), overnight at 4 °C, followed by 2 h of incubation with HRP-conjugated goat anti-rabbit (Proteintech, SA00001-2) (1:2000) at room temperature. Protein detection was performed via the use of an ultrasensitive ECL chemiluminescent substrate (Fdbio, FD8020) with a Tanon 5260Multi system. The image grayscale values were analyzed via ImageJ software.

### 2.8 Immunofluorescence (IF) staining

PC-9 cells cultured at the appropriate density (usually 70%–80% confluence) were subjected to microscopic observation, fixed with 4% paraformaldehyde for 30 min, permeabilized with drops of 0.3% Triton X-100 membrane-breaking solution for 20 min, each time for 5 min. The sections were incubated with drops of peroxidase sealing solution at room temperature and protected from light for 15 min. The sections were incubated with peroxidase blocking solution dropwise for 15 min at room temperature and protected from light and then incubated with the indicated primary antibody at 4 °C overnight, and the secondary antibody was protected from light for 30 min. The cell nuclei were stained with DAPI. Images were captured via a STELLARIS 8 DIVE laser confocal microscope + two-photon laser. The following antibodies were used for IF staining: anti-*ASPM* (1:200) and anti-*EGFR* (1:200).

### 2.9 Immunohistochemistry (IHC)

Following deparaffinization and antigen retrieval, the tissue sections were subjected to blocking and sequential incubation with primary and horseradish peroxidase (HRP)-conjugated secondary antibodies. Immunoreactivity was visualized via 3,3′-diaminobenzidine (DAB) chromogen, followed by hematoxylin counterstaining. Whole-slide images were digitally scanned via an Olympus VS200 (Yijingtong Optical Technology, Shanghai, China) slide scanner. The primary antibodies used included anti-*ASPM* (1:200).

### 2.10 Cell cycle assay

The cells were collected in Eppendorf tubes, precooled 70% ethanol was added overnight fixation, the mixture was centrifuged at 1000 *g* for 5 min, the supernatant was discarded, the prepared propidium iodide (PI) staining solution was added, the mixture was incubated for 30 min at room temperature in the dark, and the signal was detected at an excitation wavelength of 488 nm via flow cytometry.

The cells were stained with PI, and data were acquired via a MoFlo XDP Ultraflow Cytometer. The gating strategy with ModFit LT 5.0 (Verity Software House) is as follows: first, fragmentation is excluded on the basis of FSC-A/SSC-A; second, single cells are screened by FSC-H/FSC-A; and the PI-A signal is used for periodic analysis (Zhang and Lin, 2020).

### 2.11 Experimental materials

DMEM (C3113‒0500), RPMI 1640 (C3001‒0500), and fetal bovine serum (C04001‒050) were purchased from XP Biomed (Shanghai, China). Phosphate-buffered saline (PBS, G4202) and Tris-buffered saline (TBS, G0001) were purchased from Servicebio (Wuhan, China). DMSO (D4540) was purchased from Sigma‒Aldrich (Darmstadt, Germany, Merck KGaA). Trypsin-EDTA solution (0.25%, 25300054), penicillin‒streptomycin solution (15070063), Lipo3000 (L3000015) and Opti-MEM (31985070) were purchased from Thermo Fisher Scientific. siRNAs and primers were purchased from GenScript. TRIzol (9109) was purchased from Takara (Kyoto, Iapan, Takara Bio). HiScript II Q RT SuperMix for qPCR (+gDNA wiper) (223–010) and Taq Pro Universal SYBR qPCR Master Mix (Q712--02) were purchased from Vazyme. CCK8 (40203ES60) was purchased from Yeasen. The general purpose group fixative (GP24103092945) was purchased from Saiweier. Crystalline Violet (C0121) was purchased from Beyotime. AZD9291 (S7297) was purchased from Selleck. RIPA buffer (P0013C), primary antibody diluent (P0023A), skim milk powder (P0216), and Tween 20 (ST825) were purchased from Beyotime. A three-color prestained protein marker (10–250 kDa) (WJ103) was purchased from Epizyme. A 0.45 μm PVDF membrane (IPVH00010) was purchased from Millipore. A three-label four-color multiple fluorescence staining kit (AFIHC024) was purchased from AfAntibody. A cell cycle and apoptosis detection kit (No. C1052) was purchased from Beyotime.

### 2.12 Statistical analysis

**FIGURE 1:**
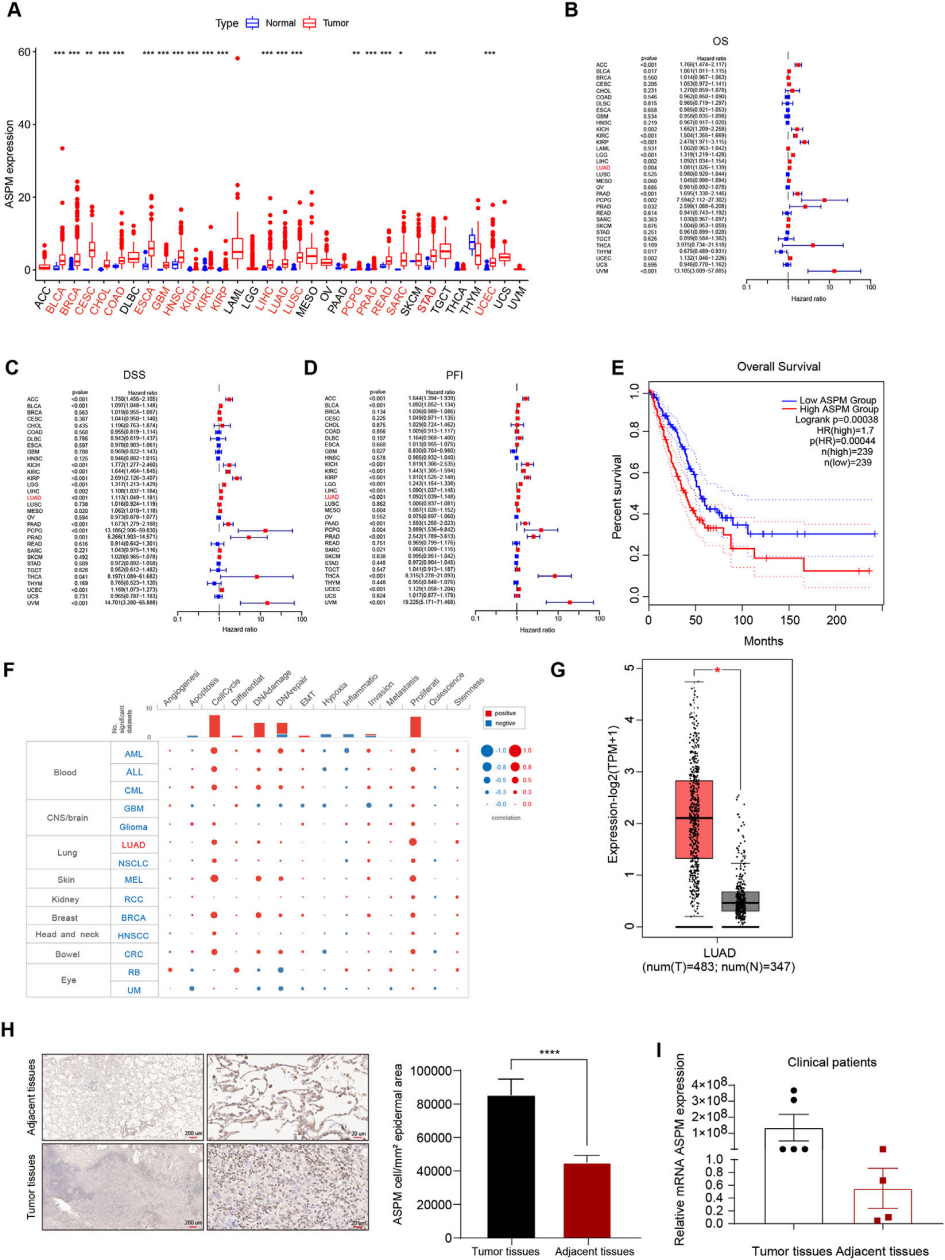
*ASPM* is highly expressed in lung cancer tissues and promotes lung cancer progression. **(A)** Box line plots of *ASPM* mRNA expression in 33 solid cancer types from the TCGA database; tumor types with significant ASPM overexpression are marked in red. The expression level of *ASPM* is denoted as FPKM. **(B)** Relationships between *ASPM* and OS. **(C)** Relationships between *ASPM* and DSS. **(D)** Relationships between *ASPM* and PFI. **(E)** Kaplan‒Meier OS curves of *ASPM* in LUAD patients from the GEPIA2 website. **(F)** Single-cell functional analysis of *ASPM* from the CancerSEA database. **(G)** mRNA expression of *ASPM* in LUAD tumor tissues versus normal tissues from the GEPIA website. **(H)** Representative images of IHC staining of lung tumor tissues as well as paraneoplastic tissue samples from patients with non-small cell lung cancer. Scale bars, 200 μm and 20 μm. The histogram represents the mean staining intensity value of the cells, and the data are expressed as the mean ± standard deviation (mean ± SD, n = 3 fields). **(I)** mRNA expression of *ASPM* in six cancer nests and matched paraneoplastic tissue samples (normalized to GAPDH; using the 2^−ΔΔCt^ method). A p value <0.05 was considered statistically significant; *p < 0.05, **p < 0.01, ***p < 0.001, ****p < 0.0001.

**FIGURE 2:**
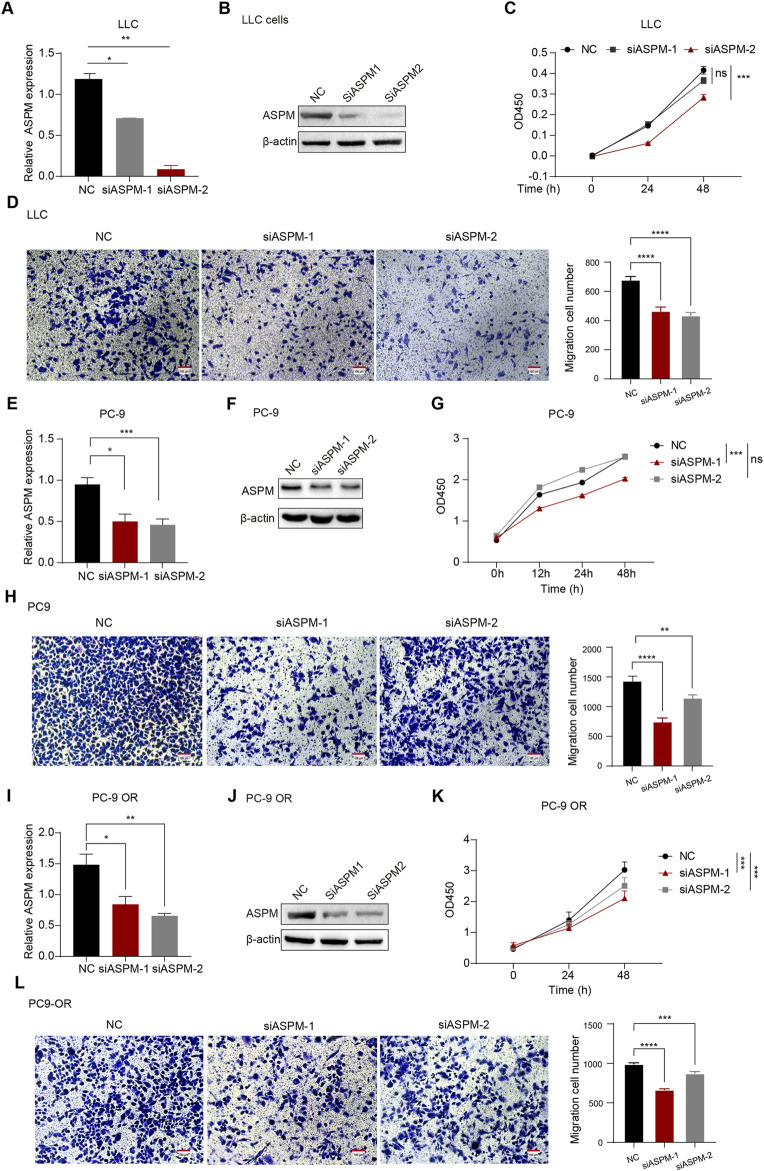
*ASPM* enhances the proliferation and invasion of lung cancer cells. **(A,E,I)** mRNA expression levels of *ASPM* were detected in LLC, PC-9, and PC-9 OR cells after silencing the *ASPM* gene. **(B,F,J)** After silencing the *ASPM* gene, the protein levels of *ASPM* were detected in LLC, PC-9, and PC-9 OR cells. **(C,G,K)** Cell viability of LLC, PC-9, and PC-9OR cells was detected via the CCK-8 assay after silencing of the *ASPM* gene. **(D, H, L)** Pictures of LLC, PC-9, and PC-9 OR cell Transwell experiments on a scale bar of 100 μm. The histogram represents the quantification of the number of cells in the field of view, and the data are presented as the means ± standard deviations (means ± SDs, n = 3 fields). *p < 0.05, **p < 0.01, ***p < 0.001, ****p < 0.0001.

**FIGURE 3:**
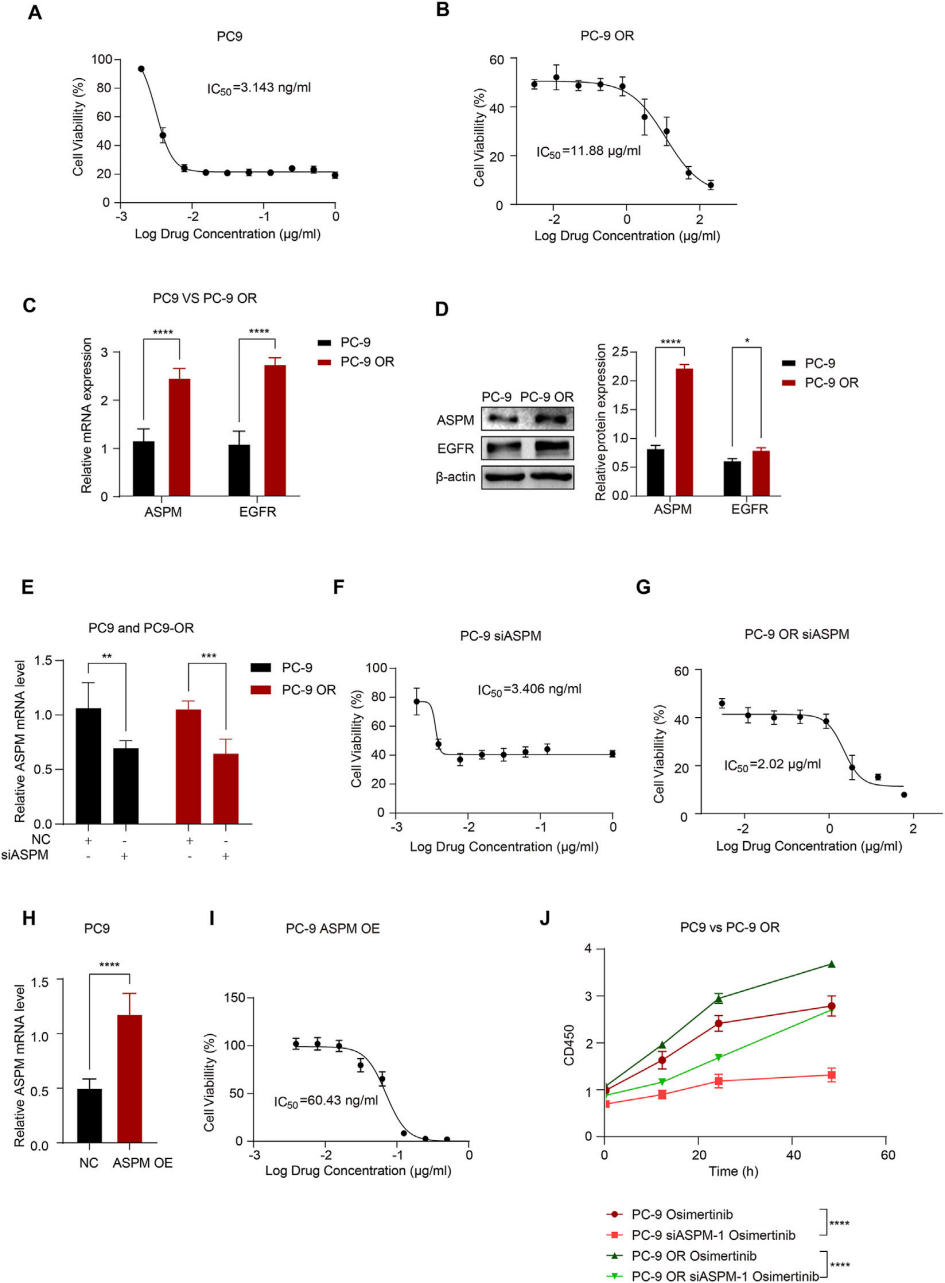
*ASPM* enhances *EGFR* resistance in lung cancer cells. **(A)** Dose‒effect relationship curves of osimertinib for PC-9 cells; **(B)** Dose‒effect relationship curves of osimertinib for PC-9 OR cells. **(C)** mRNA expression levels of *ASPM* and *EGFR* in PC-9 and PC-9 OR cells. **(D)** Protein expression of *ASPM* and *EGFR* in PC-9 and PC-9 OR cells. The histogram represents the relative gray values of the intracellular EGFR and ASPM proteins, and the data are presented as the means ± standard deviations (means ± SDs, n = 3 fields). **(E)** Silencing of the *ASPM* gene and mRNA expression levels of *ASPM* in PC-9 and PC-9 OR cells. **(F)** Dose‒effect relationship curves of osimertinib in PC-9 si*ASPM* cells. **(G)** Dose‒effect relationship curves of osimertinib in PC-9 OR si*ASPM* cells. **(H)** mRNA expression levels of *ASPM* were detected in PC-9 cells after overexpressing the *ASPM* gene. **(I)** Dose‒effect curves of osimertinib after PC-9 overexpression of *ASPM*. **(J)** The viability of the cells in each group was assessed via a CCK-8 assay. *p < 0.05, **p < 0.01, ***p < 0.001, ****p < 0.0001.

**FIGURE 4:**
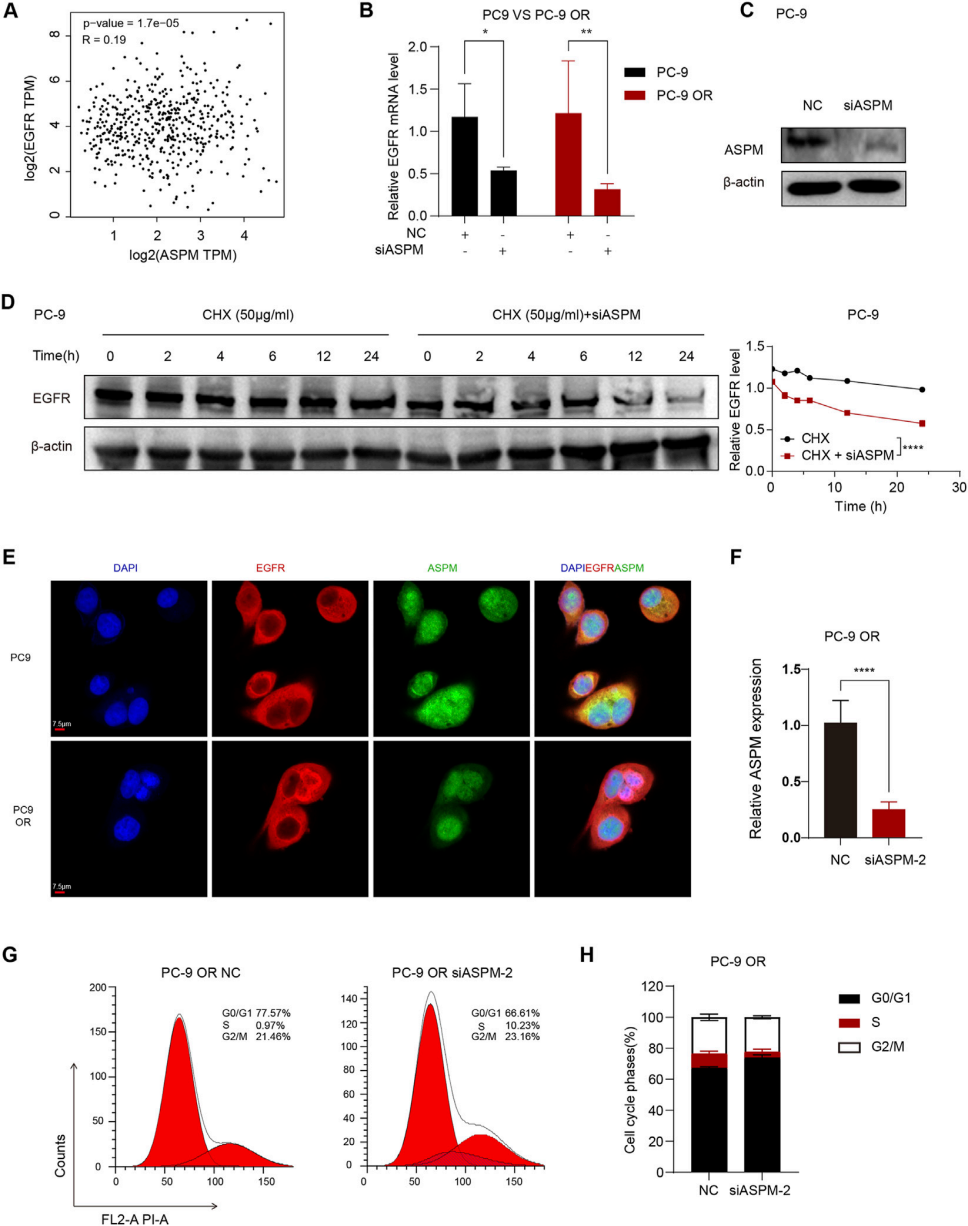
*ASPM* plays a key role in the stabilization of drug-resistant cell lines. **(A)** Scatter plot of the correlation between *ASPM* and *EGFR* expression; p < 0.05. **(B)** Silencing of *ASPM* followed by *ASPM* and *EGFR* mRNA expression. **(C)** Protein expression of *EGFR* after silencing *ASPM.* **(D)** CHX administration at various time points. The right panel represents the relative gray values of the intracellular *EGFR* proteins, and the data are presented as the means ± standard deviations (means ± SDs, n = 3 fields). **(E)** IF images of PC-9 and PC-9 OR cells incubated with primary antibodies against *EGFR* (red) and *ASPM* (green). Detection was performed using secondary antibodies coupled to Alexa Fluor 488 and Alexa Fluor 555. The cell nuclei were stained with DAPI (blue), Scale = 7.5 μm. **(F)** mRNA expression levels of *ASPM* in PC-9 OR cells after silencing of *ASPM*-2. **(G)** PC-9 OR cells in the NC group/si*ASPM*-2 group were stained with propidium iodide (PI), and the DNA content was detected via flow cytometry. The red color represents the theoretical curve that we fit via ModFit software on the basis of the DNA content distribution data of the cells. **(H)** Statistics of the flow cytometric results in H plots. n = 3, a p value <0.05 was considered statistically significant, *p < 0.05, **p < 0.01, ***p < 0.001, ****p < 0.0001.

N = 6 are shown in Figures 1I, 2A,D,H, Figures 3C,E,H, 4B. The other figures were generated from 3 independent experiments. Each set of data is presented as the mean ± standard error or mean ± standard deviation. Statistical analysis was performed via GraphPad Prism 9.5 (GraphPad software). A t-test was used to compare two independent samples, and one-way ANOVA (Tukey’s HSD test) was used to compare multiple samples. p < 0. 05 was considered statistically significant. *p < 0.05, **p < 0.01, ***p < 0.001, ****p < 0.0001.

## 3 Results

### 3.1 *ASPM* is highly expressed in lung cancer tissues and promotes lung cancer progression

Initially, we analyzed *ASPM* mRNA expression in 33 solid tumors. Twenty-one tumors had higher *ASPM* expression than did the corresponding noncancerous tissues (Figure 1A). We used Cox regression analysis to explore the tumor types affected by the *ASPM* gene, and the results revealed that overall survival (OS) was correlated with *ASPM* in 14 tumor types. For disease-specific survival (DSS), the results showed that *ASPM* also played a key role in DSS in 15 tumor types. In addition, we evaluated the progression-free interval (PFI) and confirmed that *ASPM* was a high-risk gene in 25 tumor types. *ASPM* was a high-risk gene for OS, DSS, and PFI in patients with LUAD (Figure B–D). We then investigated the predictive value of *ASPM* for survival in clinical patients with LUAD, and the results revealed that higher *ASPM* expression was significantly associated with shorter OS (Figure 1E). Next, we investigated the correlation of *ASPM* with the functional status of 14 cancers at single-cell resolution via CancerSEA. We found that ASPM was positively correlated with the cell cycle and proliferation in a variety of tumors, especially LUAD (Figure 1F). Apart of original datasets can be found in Supplementary Data Sheets 1-14, Supplementary Image 1, Supplementary Table 1. All raw data can be found at https://www.jianguoyun.com/p/DYCbnAEQ5qDRDRiOzIYGIAA.

To further verify the expression of the *ASPM* gene in LUAD, we analyzed the expression of the *ASPM* gene in LUAD cancer nests and paracancerous tissues via the GEPIA website, and the results revealed that the expression of *ASPM* in the cancer nests was much greater than that in the paracancerous tissues (Figure 1G). Furthermore, we collected six pairs of matched lung cancer tissue samples with corresponding paracancerous tissue samples, which were consistent with each other except for the differences in the diseased area and the normal area. In normal regions, other factors, such as individual patient differences, sampling time, and sample preservation conditions, remain consistent.

We used two experimental techniques, immunohistochemistry (IHC) and RT‒qPCR, to verify the expression of *ASPM* in lung cancer tissues at both the protein and RNA levels. The results of the IHC assay revealed that, compared with paracancerous tissues, lung cancer tissues presented significant positive staining for the *ASPM* protein, with a significantly greater intensity and range of staining (Figure 1H). RT‒qPCR revealed that the relative expression of *ASPM* mRNA was significantly upregulated in lung cancer tissues (Figure 1I). These results consistently indicated that the expression level of *ASPM* in lung cancer tissues was indeed significantly greater than that in the corresponding paracancerous tissues.

Taking the experimental data above into consideration, we conclude that *ASPM* plays a crucial role in the development and progression of lung cancer and that the high expression of *ASPM* in lung cancer tissues may be closely related to some malignant biological behaviors of tumor cells, such as proliferation and invasion. Therefore, *ASPM* can be targeted as an important factor affecting the development and progression of lung cancer in patients.

### 3.2 *ASPM* enhances the proliferation and invasion of lung cancer cells

To verify whether *ASPM* plays an integral role in the proliferation and invasion of relevant cell lines in lung cancer, we selected 3 cell lines: human lung cancer PC-9, PC-9 OR, and LLC. We used small interfering RNA (siRNA) to achieve transient silencing of *ASPM* in these 3 cell lines by specifically targeting two loci of the *ASPM* gene (*ASPM*-1 and *ASPM*-2). The NC group, which was transfected with a dry null scramble vector (NC), was used as a control group for comparative analysis. After silencing *ASPM*, the RT‒qPCR results revealed that *ASPM* mRNA expression in the si*ASPM*-treated group was significantly lower than that in the corresponding siNC control group in all 3 cell lines, PC-9, PC-9 OR and LLC, indicating that *ASPM* was successfully knocked down (Figures 2A,E,I). The protein levels of ASPM silencing were also verified in LLC, PC-9 and PC-9 OR cells (Figures 2B,F,J). Original strips in Supplementary Images 5, 6. We utilized these cell models to further investigate the specific role of *ASPM* in lung cancer cell proliferation and invasion.

We then used the CCK8 assay to evaluate cell growth after *ASPM* silencing. Compared with those of the control group, the OD values of the *ASPM*-silenced cells were significantly lower at all time points, which indicated that the growth of the cells significantly slowed (Figures 2C,G,K).

To investigate the effect of *ASPM* on the invasive ability of lung cancer cells, we used a Transwell assay to assess the effect of *ASPM* silencing on the invasiveness of three lung cancer cell lines (PC-9, PC-9 OR, and LLC). The results revealed that the number of invasive cells in the *ASPM*-silenced group was significantly lower than that in the null control group in all 3 cell lines (Figures 2D,H,L). These results suggest that *ASPM* silencing significantly reduces the invasive ability of these lung cancer cells. Taken together, these experimental results further confirmed that *ASPM* plays an indispensable role in the proliferation and invasion of lung cancer cells. The silencing efficiency of the *ASPM* gene was judged on the basis of the results of CCK8 cell proliferation and Transwell assays of the above three types of cells, as shown in Figure 2. If there was no special emphasis, we chose APSM-1 silencing for subsequent functional verification.

### 3.3 ASPM enhances EGFR resistance in lung cancer cells

The main mechanisms for *EGFR*-TKI resistance include *EGFR* mutations and *EGFR* overexpression (Karlsen et al., 2021; Zhou et al., 2021; Tian et al., 2022; Blaquier et al., 2023). To further investigate whether *ASPM* has any effect on the *EGFR*-TKI drug sensitivity of LUAD, we first conducted a comparative analysis of the cell growth status and metabolic activity of the PC-9 and PC-9 ORs to assess the antitumor activity of osimertinib.

In this study, we exposed PC-9 and PC-9 OR cells to different concentrations of osimertinib and observed the cellular response to the drug through cell proliferation and metabolic activity assays. The results showed that osimertinib had a significant inhibitory effect on PC-9, and the growth of PC-9 cells was effectively inhibited at lower concentrations (Figure 3A). However, PC-9 OR cells exhibited obvious resistance to osimertinib, and its IC50 was significantly greater than that of PC-9 cells, indicating that the sensitivity of PC-9 OR cells to osimertinib was greatly reduced (Figure 3B).

Next, we compared the basal mRNA and protein expression levels of *ASPM* and *EGFR* in PC-9 and PC-9 OR cells. The results revealed that the expression of *ASPM* and *EGFR* in PC-9 OR cells was significantly greater than that in PC-9 cells, which may be related to the development of cell resistance (Figures 3C,D). Original strips in Supplementary Image 4.

To further validate the effect of *ASPM* expression on the osimertinib sensitivity of lung cancer cells, we silenced the *ASPM* gene in PC-9 and PC-9 ORs (Figure 3E) to explore the role of *ASPM* in drug resistance. The IC50 was measured via the CCK8 assay, which revealed that the IC50 of PC-9 OR was significantly reduced after *ASPM* silencing. However, that of PC-9 did not change much (Figures 3F,G), suggesting that high expression of *ASPM* had a more significant effect on drug resistance. Next, we overexpressed the *ASPM* gene in PC-9 cells (Figure 3H), which have relatively low *APSM* expression. The results revealed that the sensitivity of PC-9 cells to osimertinib was greatly reduced after high expression of *ASPM* (Figure 3I). These findings further illustrate that *ASPM* plays a significant positive role in driving *EGFR*-TKI resistance in lung cancer cells through its high expression.

After *ASPM* was silenced in PC-9 and PC-9 OR cells, we measured cell proliferation through CCK8 cell experiments following the administration of osimertinib. The results showed that in both PC-9 and PC-9 OR cells, the rate of cell proliferation stimulated by osimertinib was significantly reduced after *ASPM* silencing (Figure 3J). These findings further illustrate that *ASPM* promotes drug resistance in lung cancer cells.

### 3.4 ASPM plays a key role in the stabilization of drug-resistant cell lines

Next, we explored whether there was some direct or indirect linkage between the *ASPM* gene and the *EGFR* gene in lung cancer cell lines. The scatter plot results suggested a significant correlation between the *EGFR* and *ASPM* genes (Figure 4A). Next, we performed selective gene expression analysis of PC-9 and PC-9 ORs to further explore the association of the *ASPM* gene with the *EGFR* gene. We silenced the *ASPM* gene in PC-9 and PC-9 ORs. The RT‒qPCR results revealed that when the *ASPM* gene was silenced, the expression of the *EGFR* gene appeared to be significantly decreased in both the common cell line and its drug-resistant strain (Figure 4B). There was a close connection between the *ASPM* gene and the *EGFR* gene.

The *ASPM* gene significantly maintains protein stability (Tsai et al., 2023). The stability of the EGFR protein has a positive effect on TKI resistance in lung cancer (Huang and Fu, 2015; Iyer et al., 2024). To further verify the regulatory role of *ASPM* in regulating EGFR protein stability, we silenced the *ASPM* gene and detected changes in the protein expression levels of EGFR at different time points during cyclohexanoylimide (CHX) treatment (0, 2, 4, 6, 12 and 24 h) (Chang et al., 2024). The experimental results revealed that the degradation rate of the *EGFR* protein was significantly increased in cells in which *ASPM* was knocked down (Figures 4C,D). Original strips in Supplementary Image 3. These findings suggest that the *ASPM* gene plays a crucial positive role in maintaining the stability of the EGFR protein by regulating its degradation rate. Together, these results suggest that *ASPM* plays an important role in the development of drug resistance in cells by regulating the expression of the *EGFR* gene as well as its protein stability.

To delve deeper into the effect of *ASPM* on *EGFR*-TKI resistance, we performed IF analysis on PC-9 and PC-9 ORs. By observing the intracellular distribution of ASPM and EGFR via fluorescence microscopy, the results revealed that in the PC-9 OR, the fluorescence signal of ASPM in the nucleus was significantly enhanced. These findings indicate an increase in its content in the nucleus and a higher ratio of expression in the nucleus/cytoplasm. In contrast, the fluorescent signal distribution of *ASPM* in PC-9 cells was relatively average (Figure 4E).

This difference in distribution suggests that *ASPM* may function in the nucleus and that the isoform of *ASPM*, *ASPM-2*, is localized mainly in the nucleus, where it plays an important role in cell cycle regulation (Tsai et al., 2023). As shown in Figure 2J, ASPM-2 was successfully knocked down in PC-9 OR cells. Therefore, we silenced *ASPM-2* in PC-9 OR cells (Figure 4F), and via PI/RNase staining, cell cycle assays were performed on the PC-9 OR control group and the si*ASPM-2* group. Compared with those in the control group, the percentage of PC-9 OR cells in the group with silenced *ASPM-2* stalled in the G0/G1 phase, and the percentage of cells in the G0/G1 phase significantly increased from 66.61% to 77.57% (p < 0.0001), whereas the percentage of S phase cells decreased from 10.23% to 0.97% (p < 0.0001, Figures 4G,H). Silencing *ASPM-2* causes tumor cells to stagnate in the G0/G1 phase, which can slow their proliferation. (Bai et al., 2017). *ASPM* may promote osimertinib resistance by affecting the cell cycle. These results reveal that highly expressed *ASPM* in the drug-resistant cell line PC-9 OR plays an indispensable role in maintaining the stability of *EGFR* proteins and that highly expressed *ASPM*-2 plays a key role in the cell cycle regulation and drug resistance of cancer cells, which provides a theoretical basis for the targeting of *ASPM* in the treatment of drug resistance.

## 4 Discussion

Lung cancer (Guo et al., 2021; Guo et al., 2022; Li and Qiao, 2022) is one of the most common cancers and the leading cause of cancer-related deaths globally (Ten Haaf, 2022), with non-small cell lung cancer (NSCLC) accounting for approximately 85% of all lung cancer diagnoses, and of these, lung adenocarcinoma (LUAD) (Yao et al., 2022; Zeng et al., 2023) is the most common histologic type (Hirsch et al., 2017). In recent years, owing to the increasing popularity of mutation testing for lung cancer drivers, major breakthroughs have been made in the treatment of NSCLC in the clinic—the creation of personalized, targeted treatment regimens for lung cancer patients (Fu et al., 2022). Mutations in the epidermal growth factor receptor (*EGFR*) are the most common targeted mutations in NSCLC, with *EGFR* exon 19 deletion and exon 21 Leu858Arg point mutations accounting for approximately 85% of somatic *EGFR* alterations (Castellanos et al., 2017). Although treatment with epidermal growth factor receptor tyrosine kinase inhibitors (*EGFR*-TKIs) has greatly improved remission rates and prolonged progression-free survival in *EGFR*-positive patients (Herbst et al., 2018), the majority of patients ultimately develop progressive disease (PD) after 1 year of treatment (Fu et al., 2022). In this context, increased *EGFR* expression due to bypass of the *EGFR* mutation and *EGFR* overexpression are the main reasons why patients develop *EGFR*-TKI resistance (Herbst et al., 2018; Karlsen et al., 2021). Thus, drug resistance to TKIs is an outcome that many NSCLC patients will eventually face and a direct threat to the survival of NSCLC patients (Passaro et al., 2021), coupled with the fact that there are still many deficiencies in modern medicine in terms of the understanding of NSCLC drug resistance and its treatment, and there is still a lack of effective methods for the treatment of NSCLC drug resistance (Cooper et al., 2022). Therefore, an in-depth analysis of the mechanism, identification of new therapeutic targets, and identification of methods and evidence for the clinical treatment of NSCLC are highly important.

The protein encoded by the *ASPM* gene is involved mainly in the formation and functional regulation of the spindle during cellular mitosis; specifically, *ASPM* regulates the movement of the spindle toward the poles during mitosis and maintains equal cytoplasmic division (Tsai et al., 2023). In a variety of malignant tumors, the expression level of *ASPM* is significantly elevated, which is closely related to the progression of various tumors, including prostate cancer (Pai et al., 2019), lung cancer (Cheng et al., 2023), bladder cancer (Xu et al., 2019), and kidney cancer (Deng et al., 2022).

More specifically, *ASPM*-2 can influence the biological behavior of cancer cells by increasing their cell cycle stability and interacting with the Cdk2/cell cycle protein E (Cyclin E) complex (Capecchi and Pozner, 2015). Freeman-Cook K et al. suggested that oncogenes bind to Cyclin E and develop drug resistance (Freeman-Cook et al., 2021). The interaction of Cdk2 with the cell Cyclin E complex occurs mainly during the G1 to S phase transition of the cell cycle (Chu et al., 2021).

In this study, we screened the *ASPM* gene via raw letter analysis and found that *ASPM* was highly expressed in tumor tissues and was closely associated with the survival of NSCLC patients. We selected a mouse Lewis lung cancer cell line, a human PC-9 cell line, and a PC-9 osimertinib-resistant 5-generation (PC-9 OR) cell line and verified that the *ASPM* gene inhibited cell proliferation and invasion. Furthermore, after the administration of the third-generation TKI osimertinib, we found that silencing *ASPM* significantly increased the sensitivity of drug-resistant cells to osimertinib and that the overexpression of *ASPM* also reduced the sensitivity of PC-9 cells to osimertinib. By comparing the expression of *ASPM* in PC-9 cells harboring *EGFR* 19del and its osimertinib-resistant cell line PC-9 OR, we found that the expression of *ASPM* and *EGFR* was elevated in PC-9 OR cells compared with that in PC-9 OR cells. Furthermore, the expression of *ASPM* was much greater in the nucleus than in the cytoplasm, and the silencing of *ASPM* significantly reduced the stabilization of *EGFR* proteins in the cells. However, the specific mechanism is unknown. In future research, we will focus on three aspects: whether ASPM affects the function of the EGFR ubiquitin‒proteasome pathway (e.g., E3 ligases/deubiquitinating enzymes) (Hurley and Stenmark, 2011). It may act as a chaperone (or recruit chaperone proteins) to directly maintain the conformational stability of EGFR (Zhang et al., 2022). EGFR is indirectly regulated by ASPM through transcription/translation regulation (Hildebrandt et al., 2017).

IF revealed that ASPM colocalized with EGFR in the PC-9 OR, suggesting a possible interaction between nuclear ASPM and EGFR (Zhang et al., 2022; Li et al., 2024). However, this result remains speculative owing to the lack of evidence of key molecular associations. In future experiments, we will use co-IP and PLA technology to rigorously verify whether there is a direct physical interaction between ASPM and EGFR and clarify its molecular basis and functional significance.

These findings suggest that high expression of *ASPM* in the PC-9 OR significantly enhances the protein stability of EGFR, thus leading to drug resistance. These findings reveal the pivotal role of *ASPM* in drug resistance in lung cancer cells, suggesting that inhibition of the *ASPM* gene is an important therapeutic strategy for overcoming *EGFR*-TKI resistance. Inhibiting the expression of *ASPM* may effectively reduce the proliferation of drug-resistant cells by regulating the cell cycle and weakening EGFR protein stability, which provides new ideas and methods for clinical treatment. This strategy not only helps improve the effectiveness of existing *EGFR*-TKI therapies but also may provide a theoretical basis for the development of new therapeutic options.

These findings not only increase our understanding of the mechanisms underlying the role of *ASPM* in the occurrence and development of lung cancer but also provide pivotal clues for subsequent clinical research and treatment. High expression of *ASPM* may be significantly associated with poor prognosis in lung cancer patients, and at the same time, a targeted therapeutic strategy against *ASPM* is expected to lead to the development of new effective drugs, provide more precise treatment options for lung cancer patients, inhibit tumor proliferation and invasion, prolong patient survival time and improve quality of life.

**FIGURE 5:**
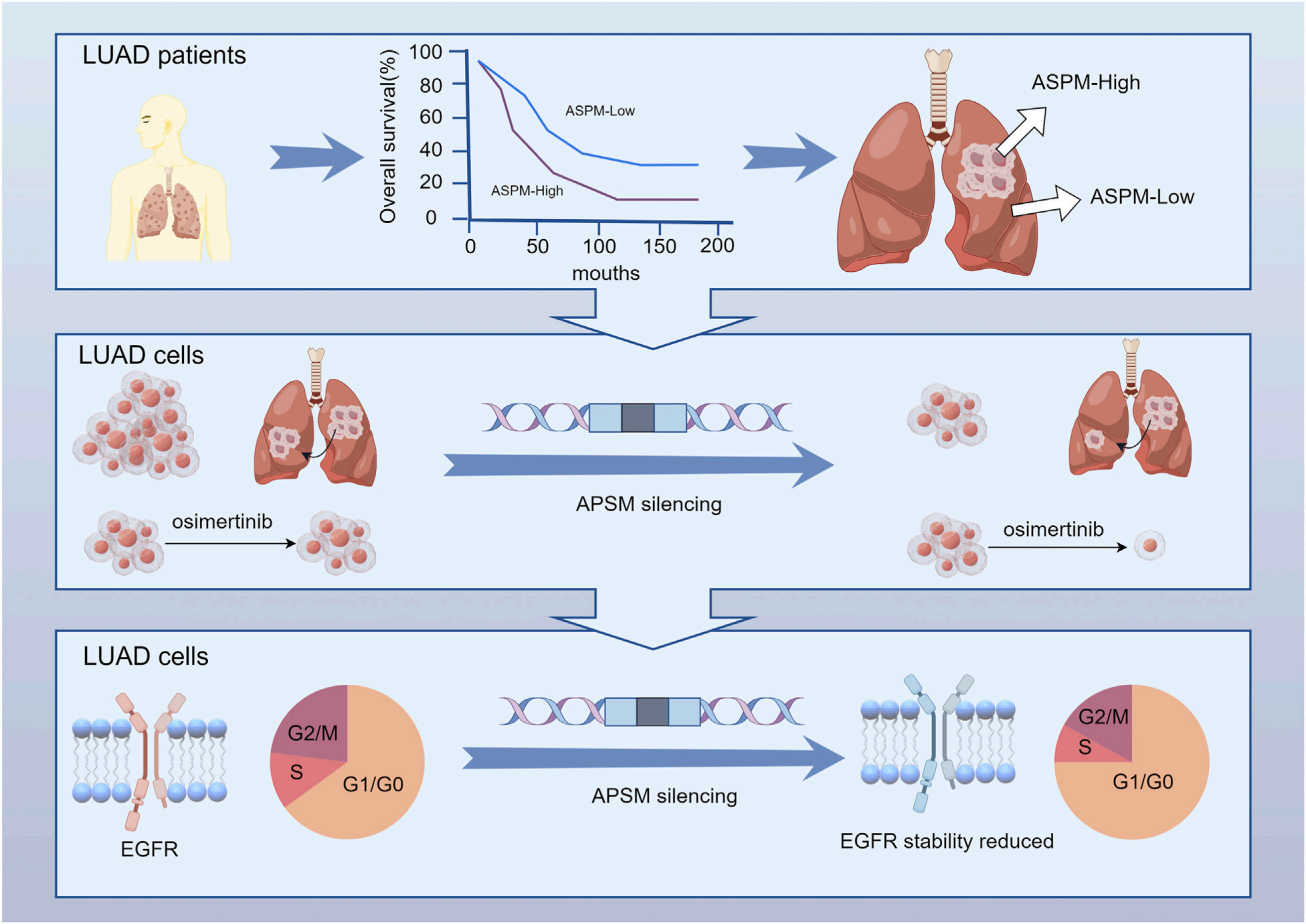
ASPM is significantly upregulated in NSCLC tumor tissues and strongly associated with reduced patient survival. ASPM silencing attenuates PC-9 and PC-9 OR malignant phenotypes, including proliferation and invasion, and sensitizes resistant cells to osimertinib. In addition, inhibiting the expression of ASPM effectively reduces damage to the cell cycle and protein stability of drug-resistant cells, thereby restoring the expression and function of EGFR.

In conclusion, despite significant progress in the treatment of NSCLC, drug resistance remains an urgent problem. In-depth parsing of drug resistance mechanisms and identification of new therapeutic targets, such as the *ASPM* gene, will provide new approaches and rationales for the clinical treatment of NSCLC (Figure 5).

Although the abnormally high expression of ASPM in drug-resistant cells suggests its value as a potential therapeutic target, there are still significant gaps in the understanding of its molecular regulatory mechanisms. Future studies need to focus on the following key scientific questions: First, through which molecular regulatory network does ASPM affect the degradation kinetics and stability maintenance of the EGFR protein (e.g., the IF data revealed strong colocalization between ASPM and EGFR mentioned in Result 4, there may be some kind of protein‒protein interaction)? Second, how do genes mediate the drug resistance phenotype of tumor cells through specific signaling pathways (e.g., the Wnt/β-catenin or PI3K/AKT pathways)? Third, we failed to use data from experiments *in vivo*. The elucidation of these mechanisms will provide a theoretical basis for the development of combination drug strategies based on ASPM-targeted therapy.

On the basis of the functional data of the cell line, such as ASPM regulation of EGFR stability and osimertinib sensitization, there is insufficient evidence for clinical translation. The lack of *in vivo* models (e.g., xenograft models with ASPM knockout) is the main limitation of this study, and the functional data of cell lines are yet to be validated *in vivo*. In conclusion, this study focused on the molecular mechanism and suggested that the ASPM-EGFR pathway is a potential target, laying the foundation for subsequent transformation.

## 5 Limitations

Although the prognostic association of ASPM is supported by public databases (Figures 1A–G), independent cohort validation was based on only six samples (Figures 1H,I), and the statistical power was limited. Expanding clinical cohort validation is a key direction for the future.

## Data Availability

The datasets presented in this study can be found in online repositories. The names of the repository/repositories and accession number(s) can be found in the article/supplementary material. All raw data can be found at https://www.jianguoyun.com/p/DYCbnAEQ5qDRDRiOzIYGIAA.

## Abbreviations

NSCLC, Non-small cell lung cancer; LUAD, lung adenocarcinoma; *EGFR,* Epidermal growth factor receptor; *EGFR*-TKIs, *EGFR* tyrosine kinase inhibitors; *ASPM,* the assembly factor for spindle microtubules; DSBs, DNA double-strand breaks; siRNA, Small interfering RNA; IF, Immunofluorescence; IHC, Immunohistochemistry.
