# Supplementary material for: Assembly factor for spindle microtubules (ASPM) promotes osimertinib resistance in lung cancer by increasing EGFR stability
Source: Front Genet. 2025 Sep 5;16:1593314. doi: 10.3389/fgene.2025.1593314 (PMC12446018; doi:10.3389/fgene.2025.1593314)

	pvalue	Hazard ratio
ACC	<0.001	1.750(1.455−2.105)
BLCA	<0.001	1.097(1.048−1.148)
BRCA	0.563	1.019(0.955−1.087)
CESC	0.387	1.041(0.950−1.140)
CHOL	0.435	1.196(0.763−1.874)
COAD	0.560	0.955(0.819−1.114)
DLBC	0.786	0.943(0.619−1.437)
ESCA	0.597	0.978(0.903−1.061)
GBM	0.708	0.969(0.822−1.143)
HNSC	0.125	0.946(0.882−1.015)
KICH	<0.001	1.772(1.277−2.460)
KIRC	<0.001	1.644(1.464−1.845)
KIRP	<0.001	2.691(2.126−3.407)
LGG	<0.001	1.317(1.213−1.429)
LIHC	0.002	1.108(1.037−1.184)
LUAD	<0.001	1.113(1.049−1.181)
LUSC	0.738	1.016(0.924−1.119)
MESO	0.020	1.062(1.010−1.118)
OV	0.594	0.973(0.878−1.077)
PAAD	<0.001	1.673(1.279−2.188)
PCPG	<0.001	13.186(2.906−59.830)
PRAD	0.001	5.266(1.903−14.571)
READ	0.616	0.914(0.642−1.301)
SARC	0.221	1.043(0.975−1.116)
SKCM	0.492	1.020(0.965−1.078)
STAD	0.509	0.972(0.892−1.058)
TGCT	0.828	0.952(0.612−1.482)
THCA	0.041	8.197(1.089−61.682)
THYM	0.169	0.765(0.523−1.120)
UCEC	<0.001	1.169(1.073−1.273)
UCS	0.731	0.965(0.787−1.183)
UVM	<0.001	14.701(3.280−65.888)

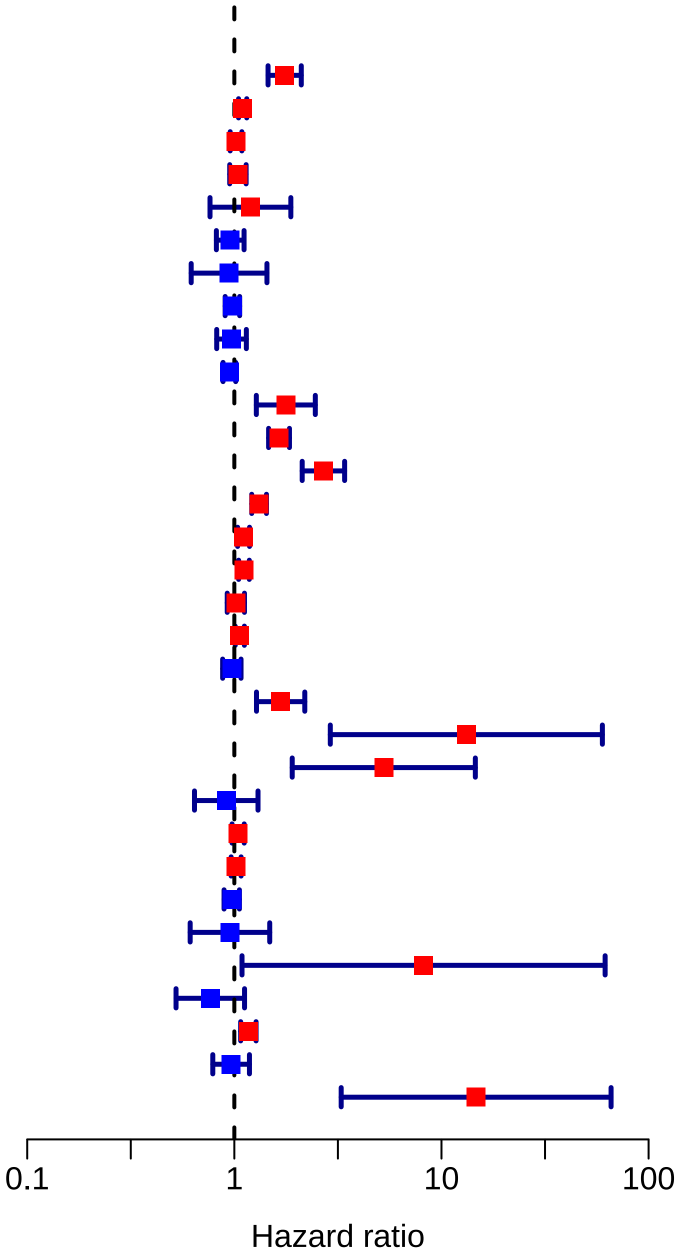

Supplement: Supplementary file 1 [file DataSheet7.pdf]
