# Supplementary figures and images for: Assembly factor for spindle microtubules (ASPM) promotes osimertinib resistance in lung cancer by increasing EGFR stability
Source: Front Genet. 2025 Sep 5;16:1593314. doi: 10.3389/fgene.2025.1593314 (PMC12446018; doi:10.3389/fgene.2025.1593314)

# Cancer: KIRP

ASPM levels + high + low

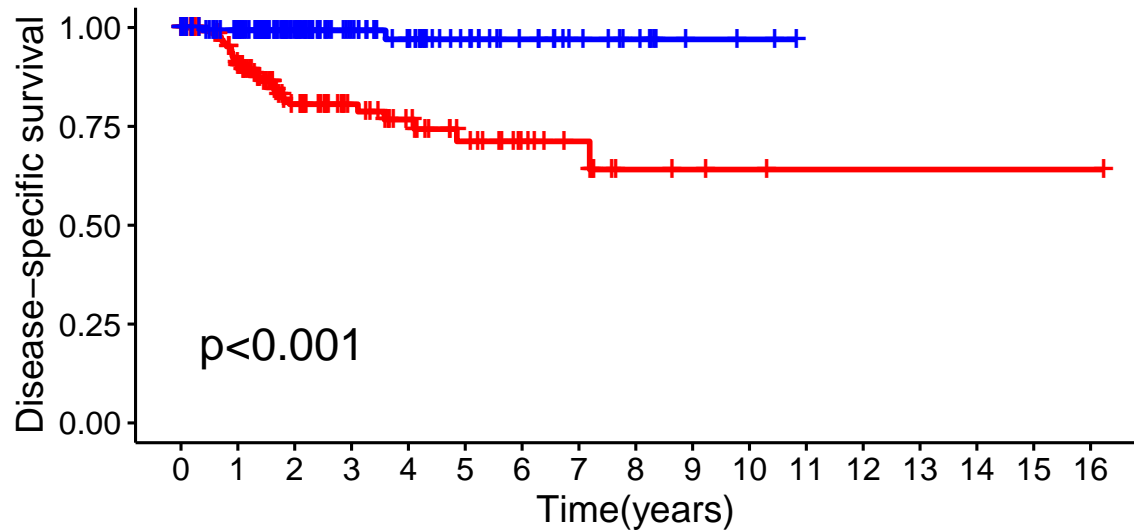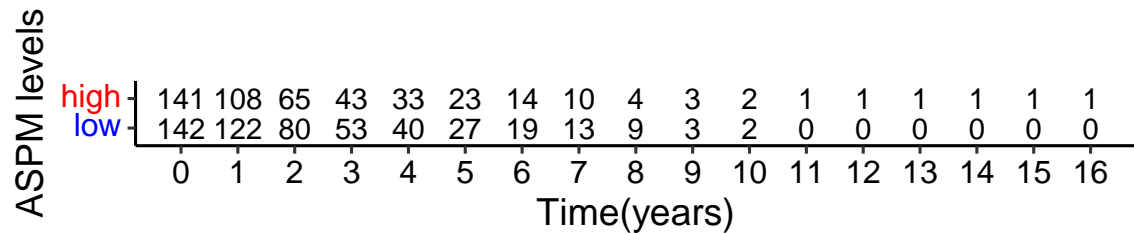

Supplement: Supplementary file 2 [file DataSheet13.pdf]

# Cancer: KIRP

ASPM levels + high + low

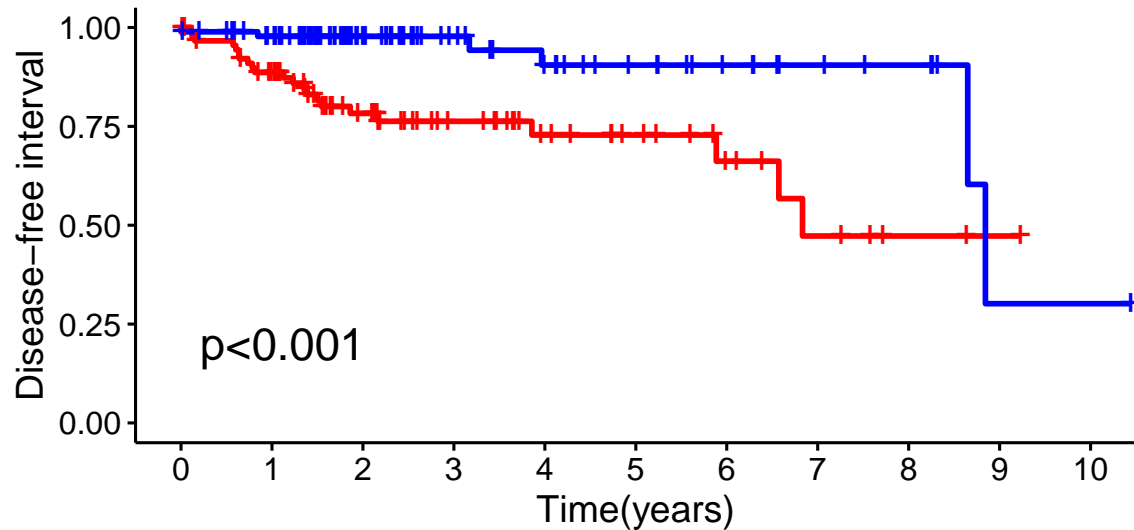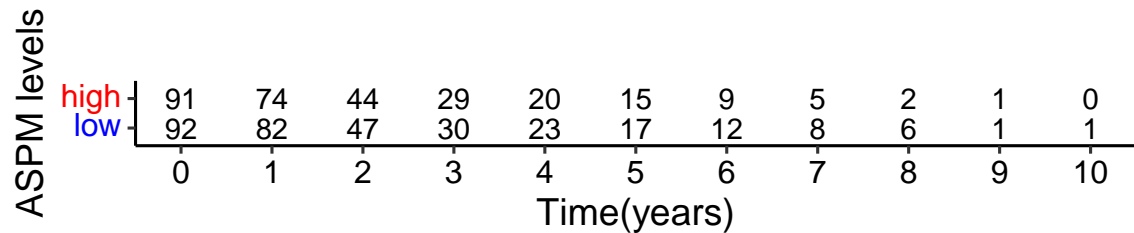

Supplement: Supplementary file 3 [file DataSheet2.pdf]

# Cancer: SARC

ASPM levels + high + low

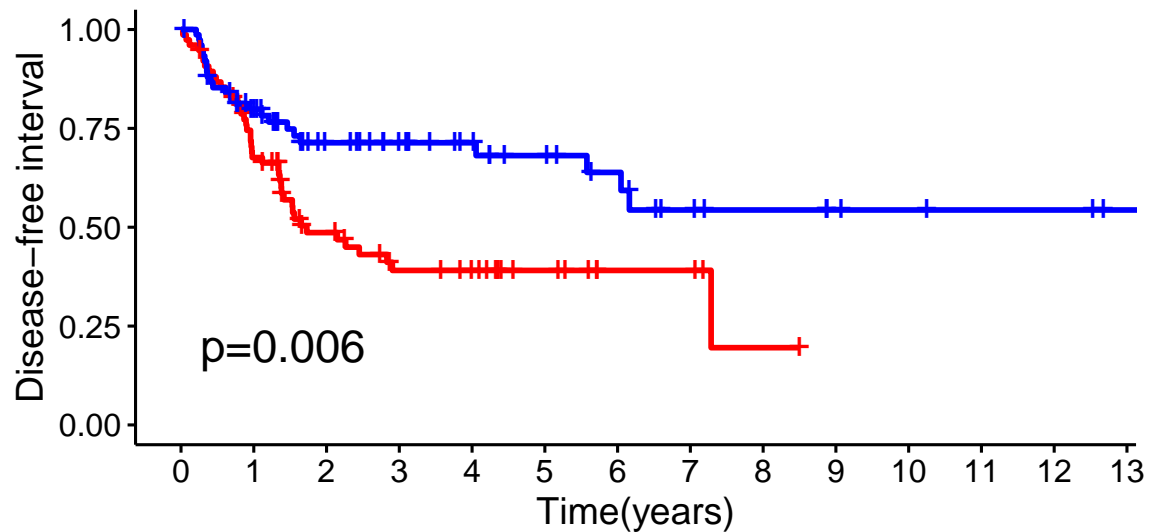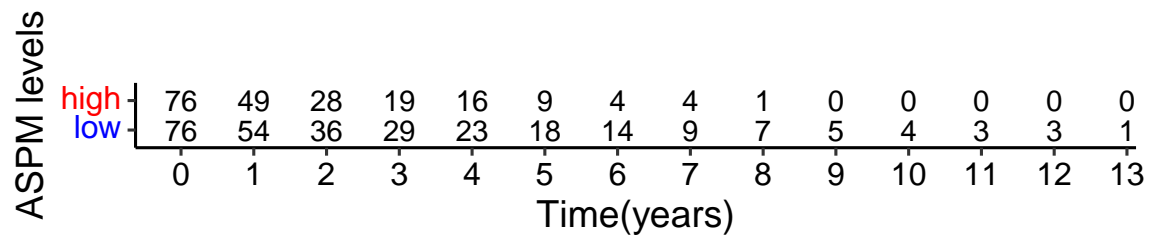

Supplement: Supplementary file 4 [file DataSheet4.pdf]

# Cancer: UCEC

ASPM levels + high + low

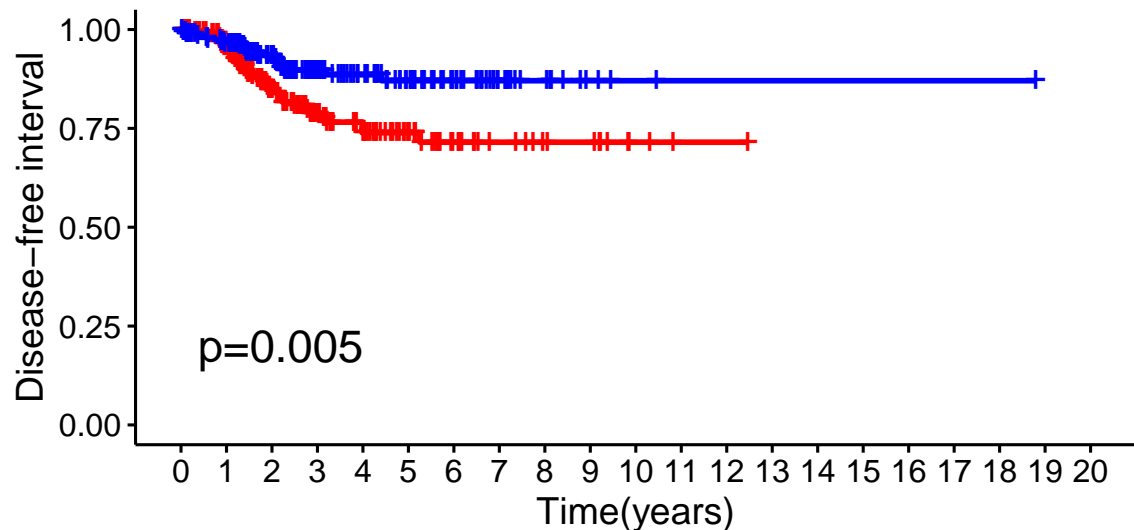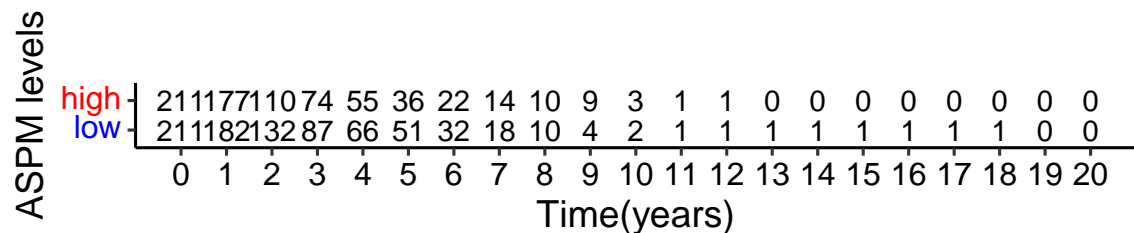

Supplement: Supplementary file 5 [file DataSheet6.pdf]

## Cancer: LGG

ASPM levels 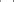 high 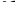 low

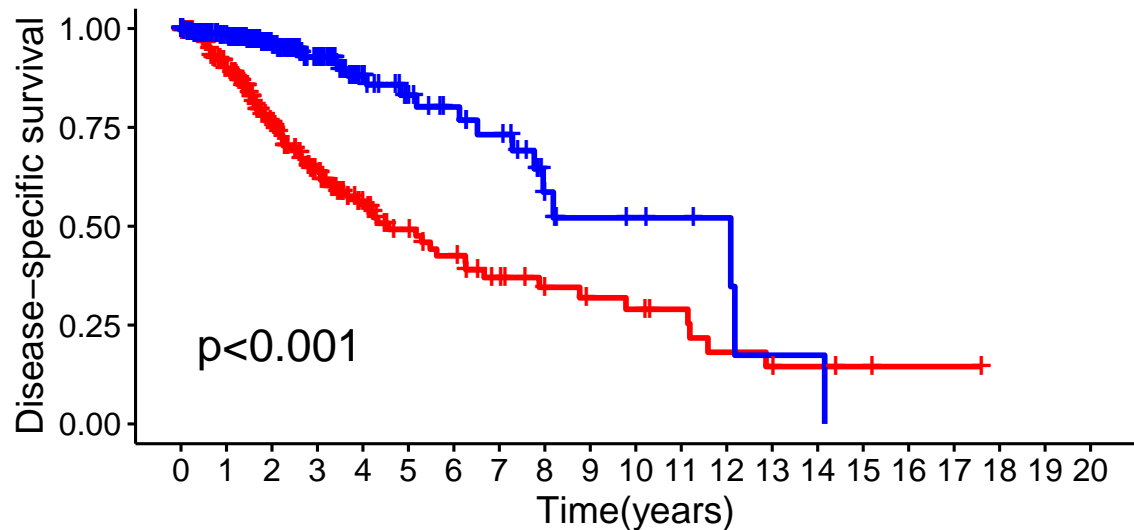

$p < 0.001$

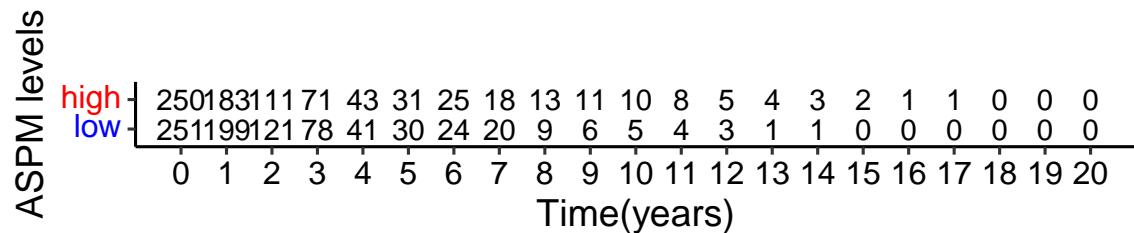

Supplement: Supplementary file 6 [file DataSheet14.pdf]

# Cancer: ACC

ASPM levels + high + low

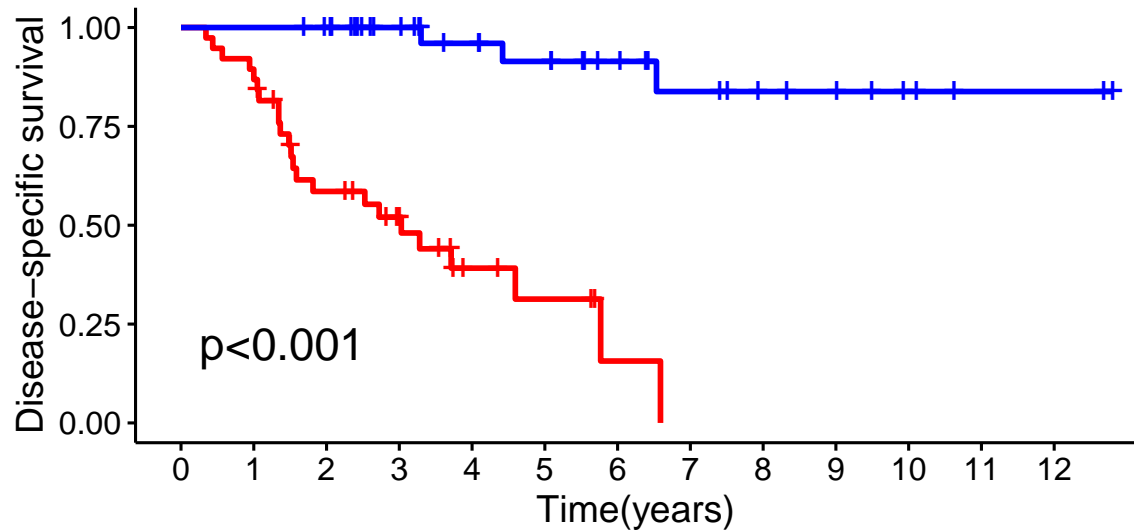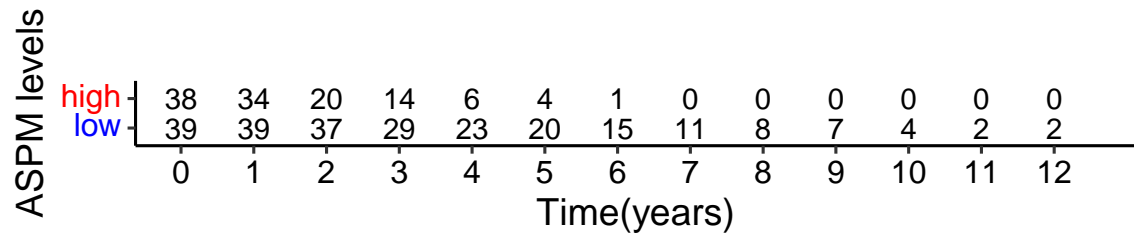

Supplement: Supplementary file 7 [file DataSheet9.pdf]

# Cancer: KICH

ASPM levels + high + low

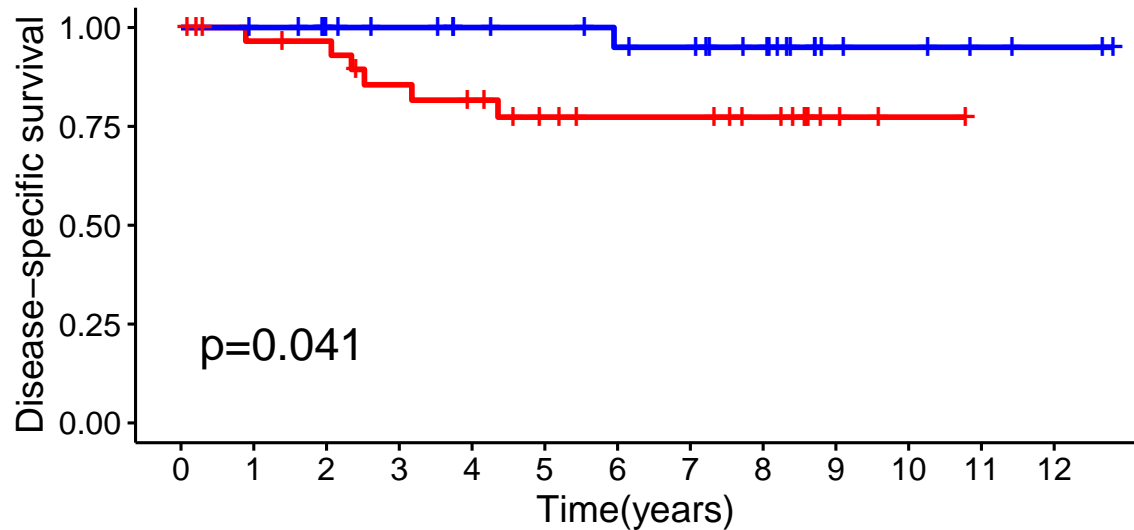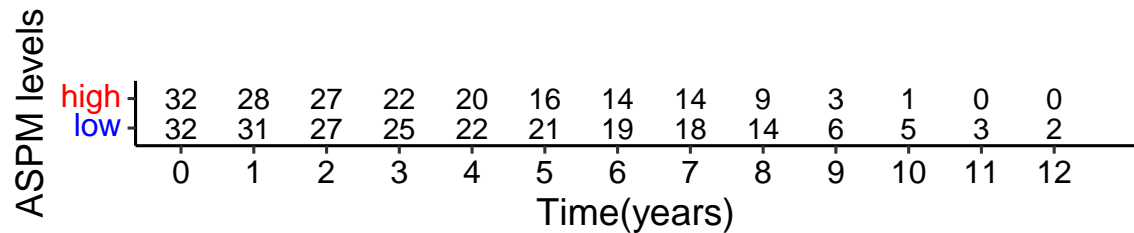

Supplement: Supplementary file 9 [file DataSheet11.pdf]

# Cancer: LIHC

ASPM levels + high + low

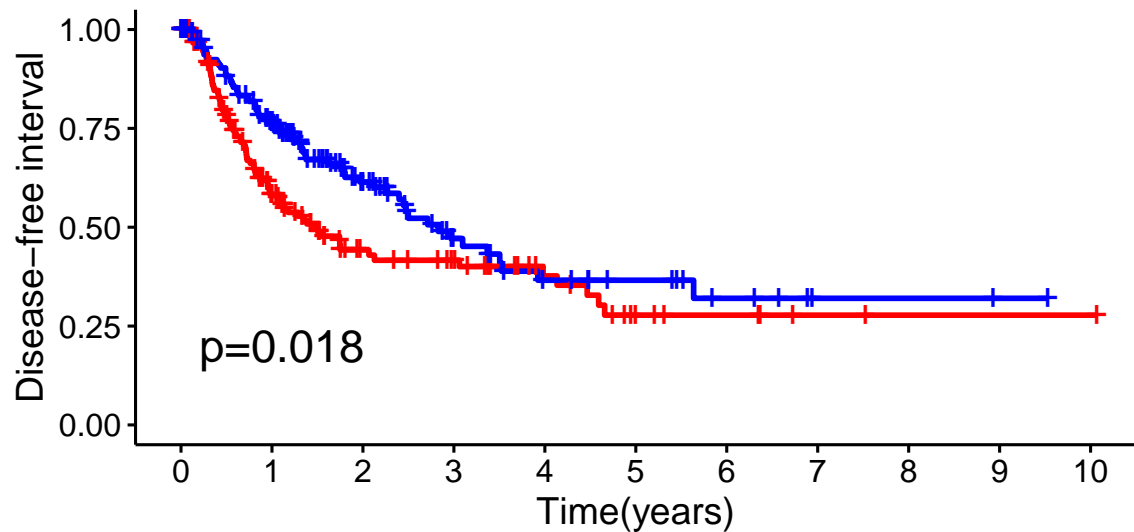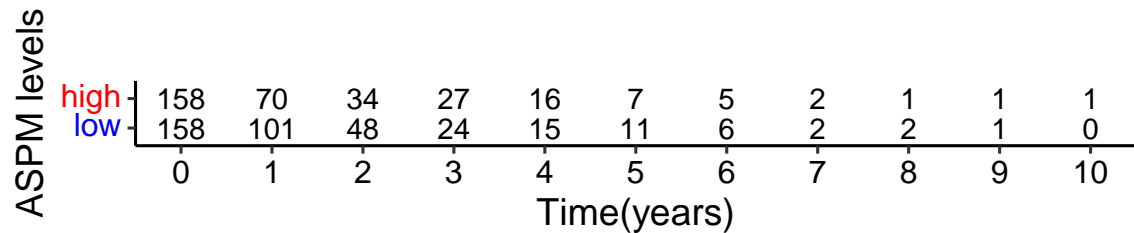

Supplement: Supplementary file 10 [file DataSheet3.pdf]

# Cancer: THCA

ASPM levels + high + low

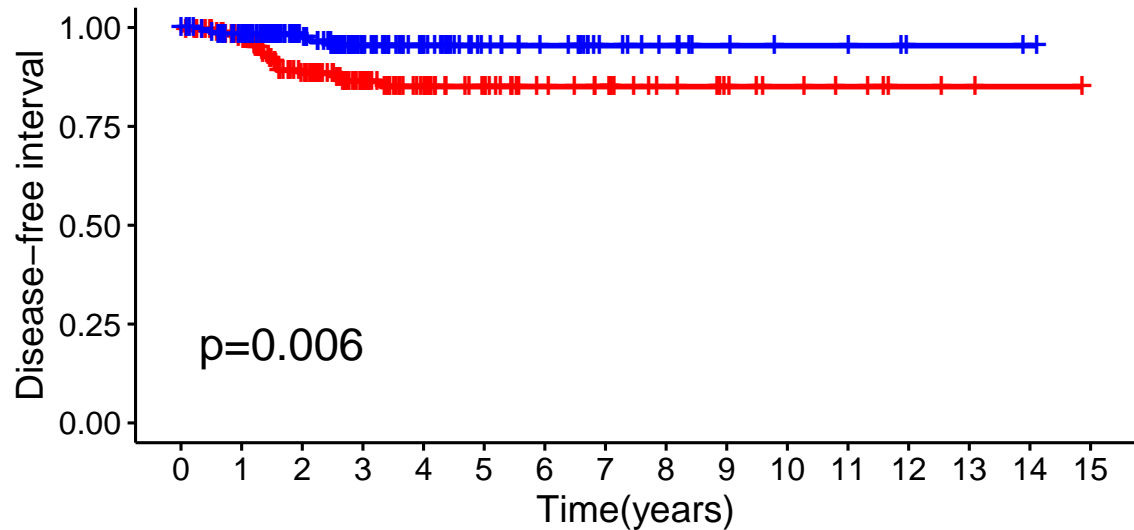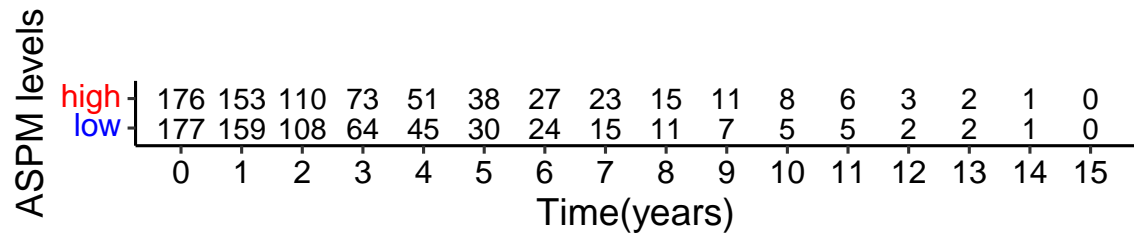

Supplement: Supplementary file 12 [file DataSheet5.pdf]

# Cancer: KIRC

ASPM levels + high + low

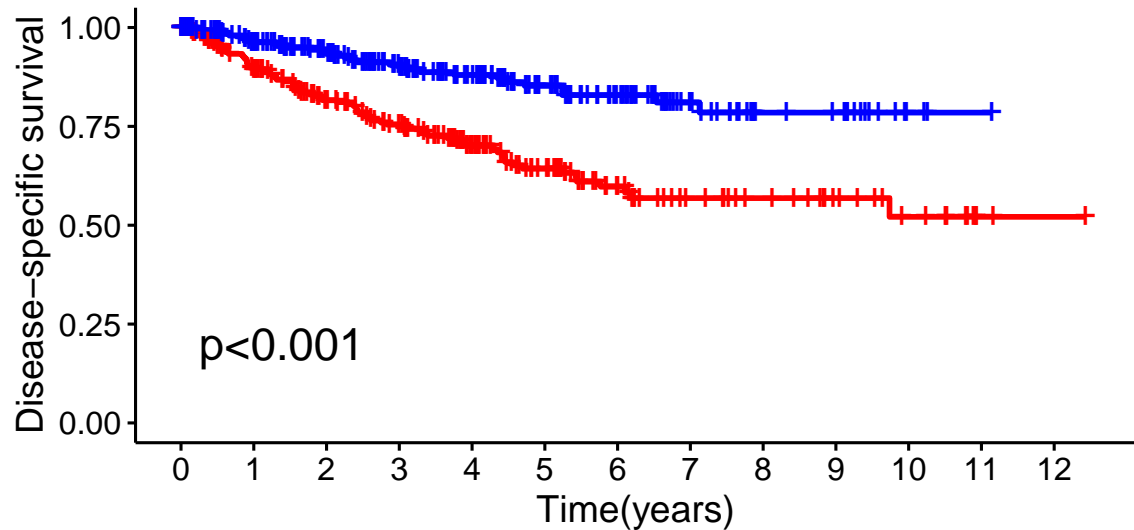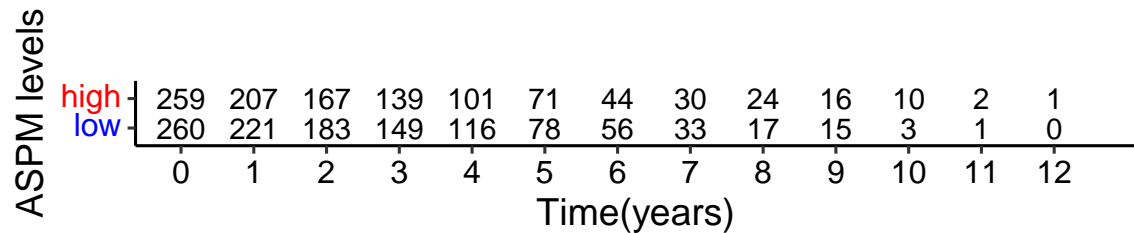

Supplement: Supplementary file 13 [file DataSheet12.pdf]

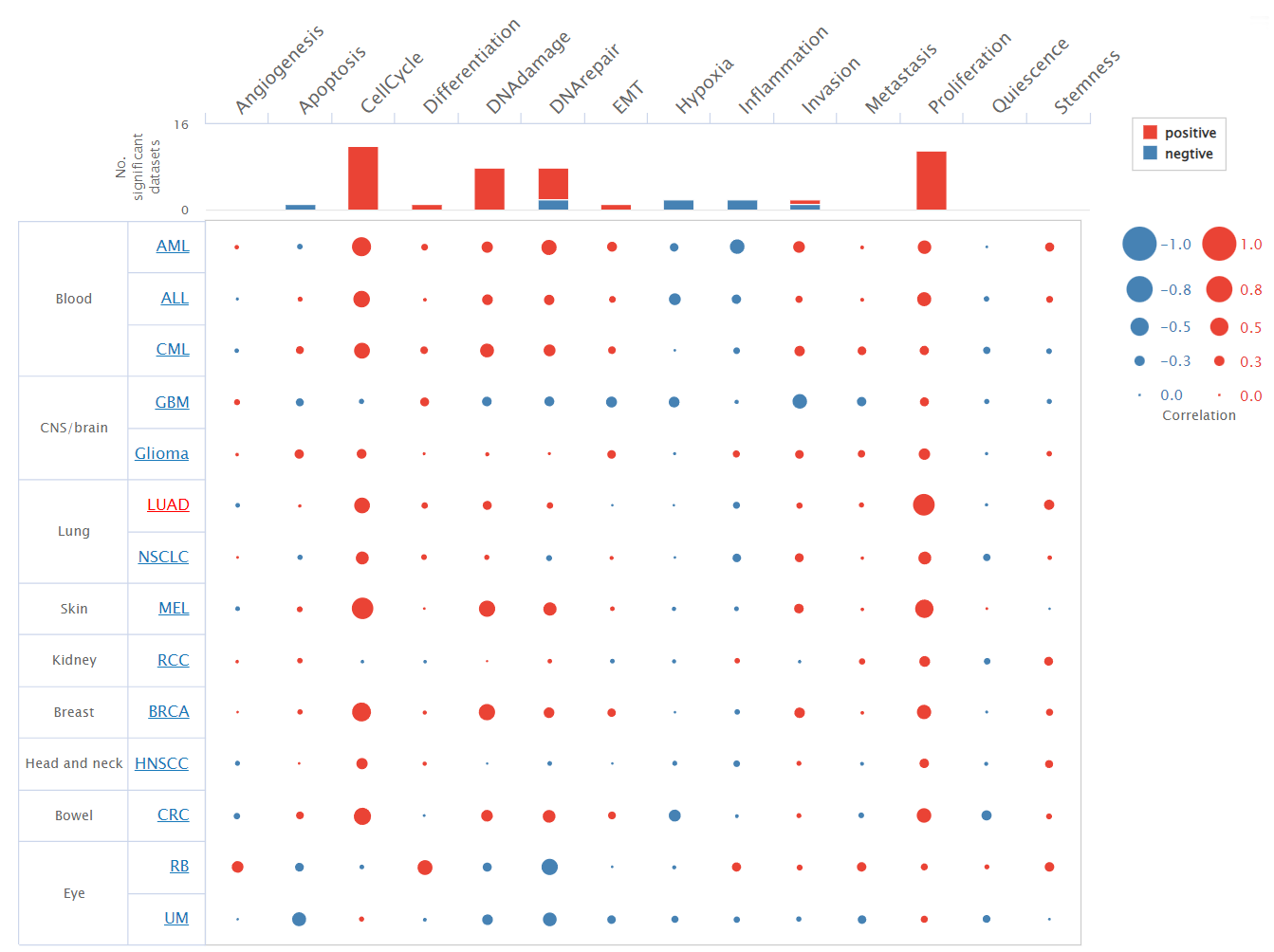

Supplement: Supplementary file 14 [file Image1.png]

# Cancer: HNSC

ASPM levels + high + low

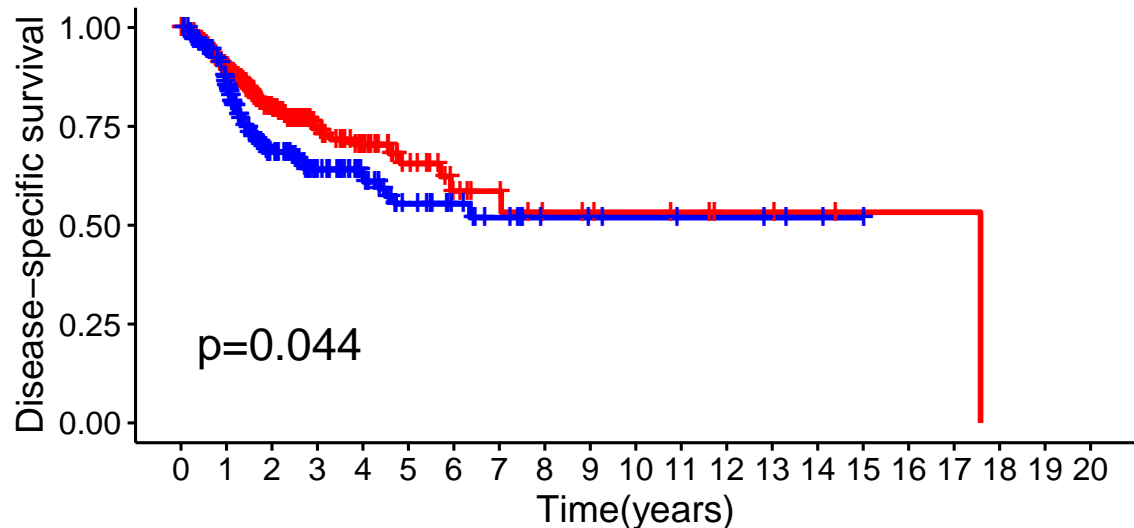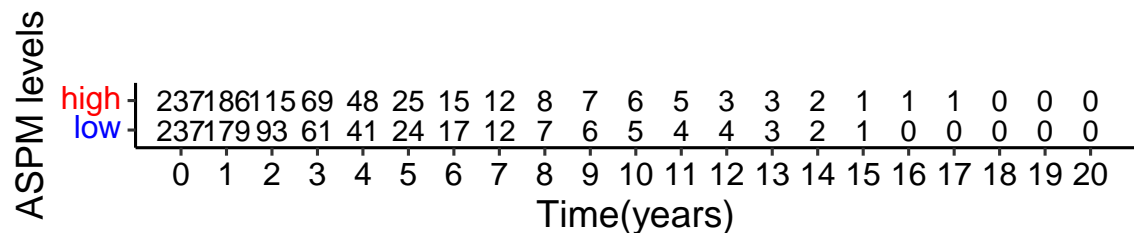

Supplement: Supplementary file 16 [file DataSheet10.pdf]
