# Supplementary material for: Assembly factor for spindle microtubules (ASPM) promotes osimertinib resistance in lung cancer by increasing EGFR stability
Source: Front Genet. 2025 Sep 5;16:1593314. doi: 10.3389/fgene.2025.1593314 (PMC12446018; doi:10.3389/fgene.2025.1593314)
Supplement: Supplementary file 8 [file Table1.docx]

ACC

BLCA BRCA CESC CHOL COAD DLBC ESCA GBM

HNSC KICH KIRC KIRP LAML LGG

LIHC

LUAD LUSC MESO OV

PAAD PCPG PRAD READ SARC SKCM STAD TGCT THCA THYM UCEC UCS

UVM

pvalue <0.001 0.017 0.560 0.205 0.231 0.546 0.815 0.658 0.534 0.219 0.002 <0.001 <0.001 0.931 <0.001 0.002 0.004 0.525 0.060 0.685 <0.001 0.002 0.032 0.614 0.363 0.876 0.251 0.626 0.109 0.017 0.002 0.595 <0.001

Hazard ratio 1.766(1.474−2.117) 1.061(1.011−1.115) 1.014(0.967−1.063) 1.053(0.972−1.141) 1.270(0.859−1.878) 0.962(0.850−1.090) 0.965(0.719−1.297) 0.985(0.921−1.053) 0.958(0.835−1.098) 0.967(0.917−1.020) 1.652(1.209−2.258) 1.504(1.355−1.669) 2.478(1.971−3.115) 1.002(0.963−1.042) 1.319(1.219−1.428) 1.092(1.034−1.154) 1.081(1.026−1.139) 0.980(0.920−1.044) 1.045(0.998−1.094) 0.981(0.892−1.078) 1.695(1.338−2.146) 7.594(2.112−27.302) 2.599(1.088−6.208) 0.941(0.743−1.192) 1.030(0.967−1.097) 1.004(0.953−1.059) 0.961(0.899−1.028) 0.899(0.584−1.382) 3.975(0.734−21.518) 0.675(0.489−0.931) 1.132(1.046−1.226) 0.946(0.770−1.162) 13.105(3.009−57.085)


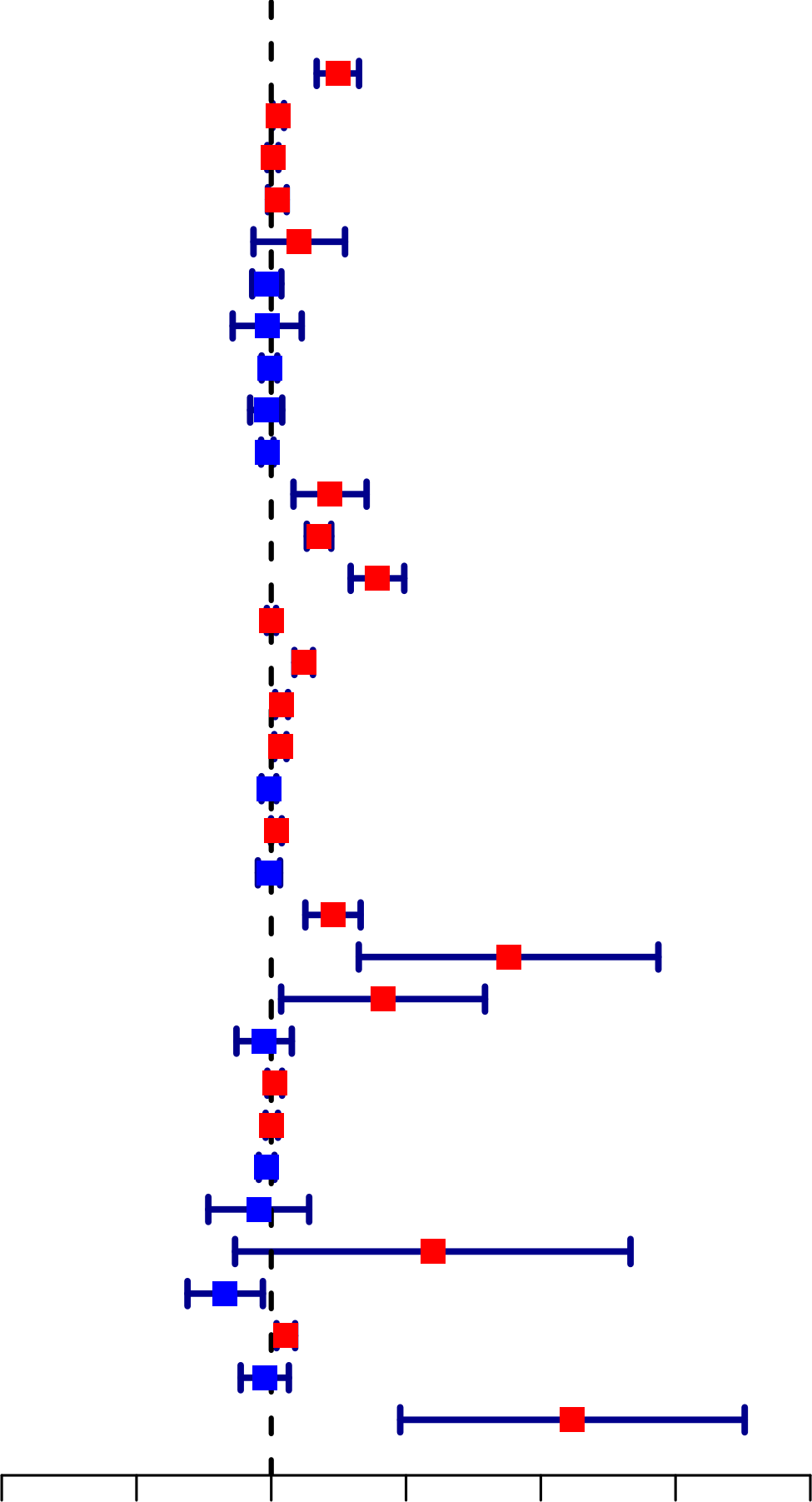


0.1 1 10 100

Hazard ratio
