# Supplementary material for: Assembly factor for spindle microtubules (ASPM) promotes osimertinib resistance in lung cancer by increasing EGFR stability
Source: Front Genet. 2025 Sep 5;16:1593314. doi: 10.3389/fgene.2025.1593314 (PMC12446018; doi:10.3389/fgene.2025.1593314)

	pvalue	Hazard ratio
ACC	0.021	4.904(1.277−18.834)
BLCA	<0.001	1.134(1.055−1.219)
BRCA	0.079	1.052(0.994−1.112)
CESC	0.196	1.096(0.954−1.261)
CHOL	0.711	0.897(0.504−1.596)
COAD	0.726	1.044(0.821−1.327)
DLBC	0.825	1.093(0.499−2.395)
ESCA	0.459	1.038(0.940−1.147)
HNSC	0.516	0.950(0.814−1.109)
KICH	0.410	0.000(0.001−4570977.217)
KIRC	0.362	1.527(0.615−3.795)
KIRP	<0.001	2.517(1.819−3.484)
LGG	0.951	0.987(0.657−1.483)
LIHC	0.003	1.086(1.028−1.147)
LUAD	0.142	1.065(0.979−1.159)
LUSC	0.138	1.078(0.976−1.190)
MESO	0.418	1.059(0.922−1.217)
OV	0.240	0.927(0.817−1.052)
PAAD	0.005	2.073(1.252−3.431)
PCPG	0.476	3.550(0.109−116.150)
PRAD	0.038	1.952(1.036−3.675)
READ	0.496	1.264(0.643−2.484)
SARC	0.039	1.076(1.004−1.154)
STAD	0.362	0.937(0.815−1.078)
TGCT	0.901	0.990(0.852−1.152)
THCA	<0.001	12.170(4.174−35.480)
UCEC	0.003	1.140(1.045−1.244)
UCS	0.178	0.612(0.299−1.251)

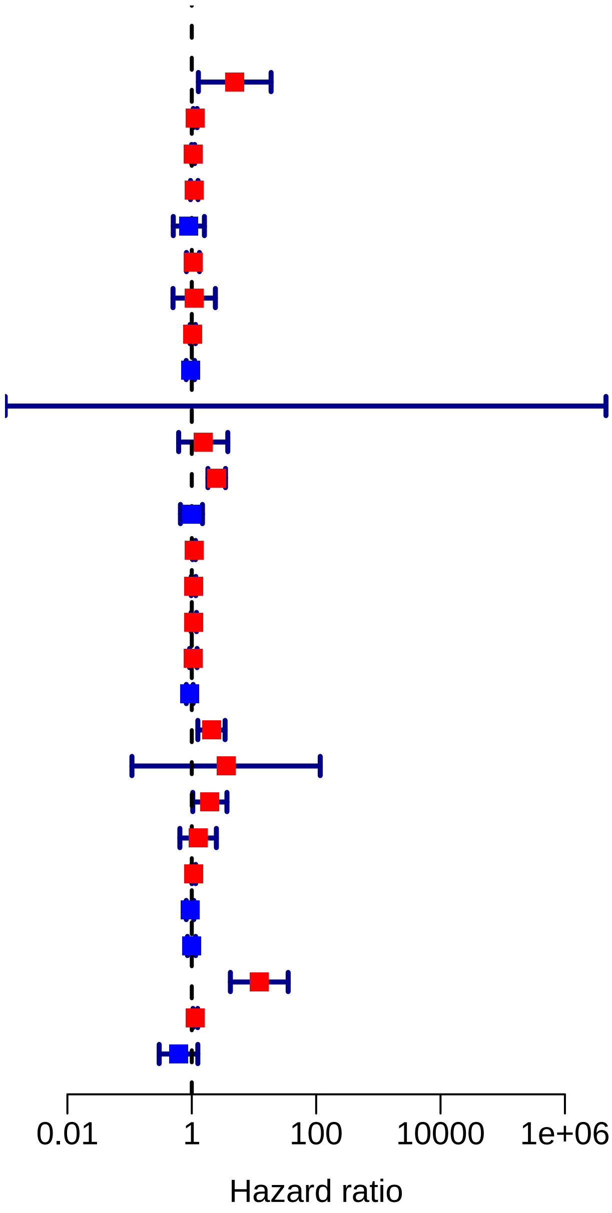

Supplement: Supplementary file 11 [file DataSheet1.pdf]
